# Cardiometabolic Risk Factor Changes Observed in Diabetes Prevention Programs in US Settings: A Systematic Review and Meta-analysis

**DOI:** 10.1371/journal.pmed.1002095

**Published:** 2016-07-26

**Authors:** Uma Mudaliar, Azadeh Zabetian, Michael Goodman, Justin B. Echouffo-Tcheugui, Ann L. Albright, Edward W. Gregg, Mohammed K. Ali

**Affiliations:** 1 Rollins School of Public Health, Emory University, Atlanta, Georgia, United States of America; 2 Division of Endocrinology, Diabetes and Hypertension, Harvard Medical School, Boston, Massachusetts, United States of America; 3 Division of Diabetes Translation, National Center for Chronic Disease Prevention and Health Promotion, US Centers for Disease Control and Prevention, Atlanta, Georgia, United States of America; University of Cambridge, UNITED KINGDOM

## Abstract

**Background:**

The Diabetes Prevention Program (DPP) study showed that weight loss in high-risk adults lowered diabetes incidence and cardiovascular disease risk. No prior analyses have aggregated weight and cardiometabolic risk factor changes observed in studies implementing DPP interventions in nonresearch settings in the United States.

**Methods and Findings:**

In this systematic review and meta-analysis, we pooled data from studies in the United States implementing DPP lifestyle modification programs (focused on modest [5%–7%] weight loss through ≥150 min of moderate physical activity per week and restriction of fat intake) in clinical, community, and online settings. We reported aggregated pre- and post-intervention weight and cardiometabolic risk factor changes (fasting blood glucose [FBG], glycosylated hemoglobin [HbA1c], systolic or diastolic blood pressure [SBP/DBP], total [TC] or HDL-cholesterol). We searched the MEDLINE, EMBASE, Cochrane Library, and Clinicaltrials.gov databases from January 1, 2003, to May 1, 2016. Two reviewers independently evaluated article eligibility and extracted data on study designs, populations enrolled, intervention program characteristics (duration, number of core and maintenance sessions), and outcomes. We used a random effects model to calculate summary estimates for each outcome and associated 95% confidence intervals (CI). To examine sources of heterogeneity, results were stratified according to the presence of maintenance sessions, risk level of participants (prediabetes or other), and intervention delivery personnel (lay or professional).

Forty-four studies that enrolled 8,995 participants met eligibility criteria. Participants had an average age of 50.8 years and body mass index (BMI) of 34.8 kg/m^2^, and 25.2% were male. On average, study follow-up was 9.3 mo (median 12.0) with a range of 1.5 to 36 months; programs offered a mean of 12.6 sessions, with mean participant attendance of 11.0 core sessions. Sixty percent of programs offered some form of post-core maintenance (either email or in person). Mean absolute changes observed were: weight -3.77 kg (95% CI: -4.55; -2.99), HbA1c -0.21% (-0.29; -0.13), FBG -2.40 mg/dL (-3.59; -1.21), SBP -4.29 mmHg (-5.73, -2.84), DBP -2.56 mmHg (-3.40, 1.71), HDL +0.85 mg/dL (-0.10, 1.60), and TC -5.34 mg/dL (-9.72, -0.97). Programs with a maintenance component achieved greater reductions in weight (additional -1.66kg) and FBG (additional -3.14 mg/dl).

Findings are subject to incomplete reporting and heterogeneity of studies included, and confounding because most included studies used pre-post study designs.

**Conclusions:**

DPP lifestyle modification programs achieved clinically meaningful weight and cardiometabolic health improvements. Together, these data suggest that additional value is gained from these programs, reinforcing that they are likely very cost-effective.

## Abstract 2

In a meta-analysis of observational and randomized studies, Uma Mudaliar and colleagues document the effects on weight and cardiovascular indicators of diet and behavior modification interventions based on the Diabetes Prevention Program.

## Author Summary

**Background:**

- In the United States and in many other countries, rates of both obesity and diabetes continue to increase every year.
- The US Diabetes Prevention Program (DPP) trial studied people with a high risk of diabetes and placed them in a program encouraging adoption of both a healthy diet and exercise, consisting of 16 core sessions with ongoing support afterwards.
- The DPP trial showed that such a program could decrease the incidence of diabetes and that it helped people lose weight and decrease their cardiovascular risk factors.

**Why Was This Study Done?:**

- This study was done to collect all of the available data from programs based on implementation of the DPP in different settings and to combine them into a large analysis to understand whether such programs were associated with weight, blood sugar, blood pressure, and cholesterol.
- Our study was designed specifically to find out if the programs worked, which health parameters changed and which program characteristics were the most useful.

**What Did the Researchers Do and Find?:**

- We performed a systematic review and meta-analysis by looking at four different databases of articles and selecting all of those which used principles from the DPP.
- We identified 44 studies with a total of 8,995 participants who were enrolled in different versions of the DPP.
- These programs differed in many ways from the original DPP trial—they were delivered in different settings, had different providers, and involved group sessions rather than individual sessions.
- We found that even with these modifications, the programs were still associated with favorable changes in weight, blood pressure, cholesterol and blood sugar.

**What Do These Findings Mean?:**

- Programs based on the DPP have the potential to help a large number of people in the community to lose weight, decrease their risk of diabetes, and help improve other markers of health.
- These programs could also be a cost-effective way to help prevent diabetes and help people live healthier lives.

## Introduction

Diabetes currently affects approximately 9.3% of the United States population [1], and by 2050, its prevalence is expected to reach 25% [2]. Adults with diabetes have two to four times higher rates of death from heart disease or stroke, and they have medical expenses that are more than two times higher than those for people without diabetes [3–6]. The total annual economic burden associated with diabetes was US$245 billion in 2012, with US$176 billion incurred as direct medical expenditures [3]. In addition, 86 million US adults (35% of the population) have prediabetes [1], which puts them at over four times the risk of progressing to diabetes compared to those who are normoglycemic [7,8].

Large randomized controlled studies [9–11], including the US Diabetes Prevention Program (DPP) trial, have shown that intensive and structured lifestyle modification interventions in people with impaired glucose tolerance can lower the incidence of diabetes by 30%–58% compared to basic lifestyle advice [10–15]. Although primary prevention of diabetes through lifestyle changes is deemed cost-effective, the first-year cost of delivering the original DPP lifestyle intervention was prohibitive (US$1,399 per participant) [16–18]. In addition, lifestyle and cultural patterns vary significantly, across and even within communities, necessitating tailoring of interventions according to regional and ethnic differences to achieve effectiveness, acceptability, and sustainability [19]. To find acceptable, lower-cost alternatives to the resource-intensive DPP lifestyle interventions, a number of studies tested adaptations of DPP delivery in typical US clinics and communities, but still retained the DPP’s core principles of modest weight loss, calorie-restricted diets, and 150 min of moderate-intensity exercise per week; on average, these DPP lifestyle interventions were associated with meaningful pre-post weight loss of approximately 4% [20]. However, it remains unknown whether these nonresearch lifestyle intervention programs were associated with meaningful changes in glycemic markers (fasting blood glucose [FBG] and glycosylated hemoglobin [HbA1c]), blood pressure (BP), and lipids (high density lipoprotein [HDL] and total cholesterol [TC]). Furthermore, there are no comparisons of how data from these translation or effectiveness studies compare to metabolic changes observed in the DPP efficacy trial itself [20,21]. It also remains unclear whether benefits of lifestyle modification programs for diabetes prevention are equally beneficial across persons with objectively defined prediabetes versus those identified only through risk factors for diabetes (e.g., being overweight and hypertensive), and whether program characteristics (e.g., presence of a maintenance component or type of provider) are associated with better outcomes.

## Methods

### Study Selection

We systematically searched four electronic databases (MEDLINE, EMBASE, Cochrane Library, and Clinicaltrials.gov) for translation or effectiveness studies that tested delivery adaptations of DPP-lifestyle principles in the US and were published between January 1, 2003, and May 1, 2016. Including studies with a similar exposure (DPP-lifestyle principles), albeit via different delivery modalities, and similar outcome measurements allows for the heterogeneity in these studies to answer questions regarding external validity that the original study could not. Indeed, the focus of this work was to inform wider implementation and scaling of interventions in the US.

The search terms used are listed in S1 Table. We supplemented our searches by hand searching reference lists of included articles and other reviews of this topic [22].

We included studies if they met three eligibility criteria. First, each study needed to evaluate implementation of lifestyle intervention programs based on tested DPP principles in US settings. Second, the studies had to have reported pre- and post-intervention estimates for at least one of the following measures: weight, HbA1c, FBG (venous or capillary), systolic or diastolic BP (SBP, DBP), HDL, or TC. Finally, each eligible study had to include adults (age ≥18) at high risk of developing diabetes. To qualify as “high risk,” the target population could have either of the following criteria:

1 A diagnosis of prediabetes [23,24]. This could be a self-report of a previous medical diagnosis, or by blood glucose testing, such as
  a FBG ≥100 mg/dl (impaired fasting glucose [IFG]) or
  b 2 h post-challenge glucose of 140–199 mg/dl (impaired glucose tolerance [IGT]) or
  c Random (nonfasting) blood glucose between 110–199 mg/dl) [25]; or
  d HbA1c of 5.7%–6.4% [24]

OR

2 Presence of risk factors for diabetes:
  a Body mass index (BMI) ≥25 kg/m^2^ AND one additional risk factor such as previous gestational diabetes (GDM), family history, or minority race/ethnicity such as being Asian American, Hispanic, or non-Hispanic US blacks [25]

OR

b An American Diabetes Association diabetes risk score of greater than 5 [26]

This systematic review and meta-analysis was focused on studies in the US that implemented specific principles tested in the DPP. The study needed to specifically state that it was DPP inspired, and have both an exercise and dietary component in its intervention. We excluded studies with children, adolescents, or lifestyle interventions that did not involve combined diet and exercise principles tested in the DPP trial. If participants had polycystic ovarian syndrome, current or recent pregnancy, or tested the use of medications (such as metformin) to prevent diabetes, these studies were excluded. If studies had a majority of prediabetes participants (greater than 50%), we included these studies in order to be able to include all available data.

Abstracts were reviewed independently by two authors (UM and AZ) who used the criteria above to determine study eligibility. All discrepancies were resolved by consensus with a third study author (MKA.) When necessary information was not reported in the study, authors of the original article were contacted for further details.

Data regarding study population characteristics, study designs, characteristics of interventions tested (number of core and maintenance sessions, duration of interventions, and follow up time periods), and on baseline and follow-up values for each outcome (weight, FBG, HbA1c, SBP, DBP, HDL, and TC) were extracted by one author (UM) from each eligible article.

### Quality Assessment

The DPP trial findings established that standard health advice alone had a low level of efficacy in reducing diabetes incidence among overweight participants with biochemically confirmed impaired glucose tolerance and elevated fasting glucose [13]. Structured behavioral modification interventions, adherence to rigorous lifestyle principles, and associated weight loss were shown to be effective [27]. Unlike large trials such as the DPP, translation studies tend to be small and often use quasi-experimental designs (e.g., single group pre- and post- intervention or pre-post evaluations with control groups) in which random allocation and blinding are impossible [13]. To assess study quality and facilitate interpretation of the available literature, we applied a scoring system with three criteria, each contributing one point in the criteria adapted from those proposed by Juni et al. [28]. The first criterion assessed whether studies used any steps to minimize attrition bias by using an intention to treat analysis, achieving low attrition rates (≤20%), or by comparing characteristics of completers and noncompleters. The second criterion was to assign higher quality to studies that included a control group (randomized, matched, or unmatched comparison). The third criterion focused on whether the study reported on four or more of the following aspects of translating evidence: describing the process of designing the program, describing the enrollment process, documenting session attendance, reporting costs and/or resource inputs, documenting the training process or qualifications of personnel, or describing the qualitative feedback from participants or providers. The latter was considered an important aspect of study quality given that studies were included based on their ability to provide information about how DPP lifestyle programs were implemented and how successful they were. These quality assessment criteria were chosen because they support study replication and comparison. A study was categorized as “high quality” if it had at least two out of three possible points. Further details are provided in the S3 Table.

### Statistical Analysis

Using pooled data, descriptive characteristics of the study populations were calculated as weighted means based on sample size. Since very few of the studies had comparison groups, the intervention arms of controlled studies were treated as pre-post groups and aggregated with the remaining single-group pre-post studies. When studies included multiple intervention arms, each distinct intervention arm was treated as a separate pre-post group. For example, in studies in which a lifestyle intervention was delivered using two different media (in person versus remote), these counted as two separate intervention groups within the same study.

Estimates of pre-post intervention change in each outcome and the corresponding standard errors were obtained or calculated based on the data reported. To standardize the length of follow-up, we focused on the 1 y interval between pre- and post- intervention measures. If a study conducted several follow-up assessments for a given outcome, the reported value at the time point closest to 1 y was used to calculate the change from the baseline. We used random effects meta-analysis techniques to calculate aggregate estimates and measures of dispersion to account for inter-study heterogeneity. All estimates were accompanied by a χ^2^ test for heterogeneity with a corresponding I^2^ value.

To explore what program characteristics may have been associated with risk factor changes, we performed stratified analyses according to delivery format (individual or group), intervention delivery personnel (by health professionals, lay community center staff, or electronic media), location of the intervention (clinical or community setting), and inclusion of a maintenance component in the study protocol (yes or no). When there was more than one type of delivery format, the delivery format of the core sessions took precedence. For example, if an article had core sessions in the community and a remote component for maintenance, this was coded as “community.” Stratified analyses were conducted for each outcome, except HbA1c, for which data were insufficient to allow stratification. We also stratified by the method used for risk classification (blood glucose criteria or risk factor criteria to classify as “high-risk”). We tested whether any program characteristics were associated with weight change using Spearman’s rank correlation analyses. Program characteristics of interest, in addition to those listed above, included the mean number and duration of core sessions offered, average participant age and BMI, proportions of males and non-Hispanic whites (NHWs) in the study sample, and attrition. Lastly, we evaluated aggregate outcomes in high versus average quality studies.

When studies included a control arm, we included them in separate analyses of the intervention arms against the control arms to determine what incremental changes in weight and cardiometabolic risks was observed.

All calculations were carried out using MIX 2.0 statistical software [29]. Statistically significant differences were noted based on a conservative definition of non-overlapping 95% confidence intervals. All findings were reported in reference to the 1 y findings reported by the original DPP trial [13].

## Results

### Study and Participant Characteristics

**Fig 1:**
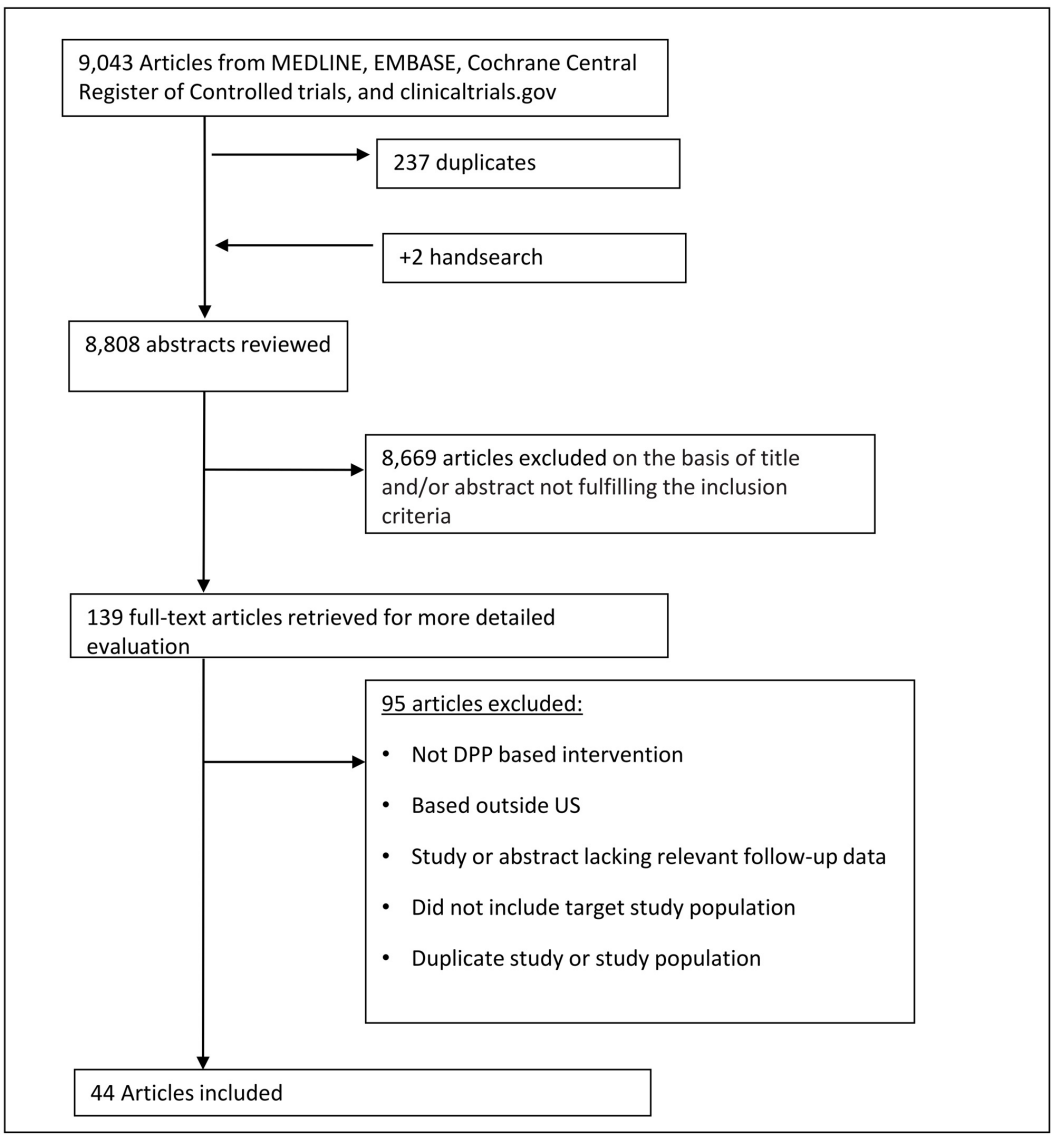
Flow diagram of study search. This flow chart describes the number of studies that were involved in each step of the process of study selection, from the initial study search. After the application of inclusion and exclusion criteria, 44 studies met criteria and were included in the final analysis.

A total of 44 studies, which included 48 intervention groups, met eligibility for inclusion. Of these, 22 studies were single group pre-post designs [30–51], 3 studies had two intervention arms (each contributing two separate groups for analysis) [52–54], 18 studies had separate control arms [55–72], and 1 study had three arms—two intervention groups and one control [73]. Data on weight change from baseline were available from all intervention groups. There were 21 groups that had pre- and post-intervention data on FBG, 8 had HbA1c measures, 23 had SBP measures, 22 had DBP measures, 14 had HDL measures, and 12 had TC measures. Further details are listed in Fig 1.

A total of 8,995 participants were enrolled across all intervention arms. Aggregate participant demographic and clinical characteristics were similar to participants enrolled in the original DPP study (Table 1). At enrollment, participants’ mean age was 50.8 y, mean weight was 99.3 kg, mean BMI was 34.8 kg/m^2^, 25.2% were male, and 32.9% were non-Hispanic white. Mean baseline levels of cardiovascular risk factors were: 5.9% (HbA1c), 104.6 mg/dl (FBG), 128.7/79.5 mmHg (SBP/DBP), 46.1 mg/dl (HDL), and 183.7 mg/dl (TC). Of the 48 intervention groups, 18 used blood glucose measures to classify risk for diabetes, 22 used the presence of risk factors, and 8 included participants defined as high risk by either of the above criteria.

**Table 1:** Baseline characteristics of trials (aggregate) versus DPP.

	DPP trial (lifestyle arm)	Included trials (IQR)
Age (years)	50.6	50.8 (49.0–56.4)
BMI	33.7	34.8 (32.2–36.6)
Male (%)	32.0	25.2% (15.0–31.3)
NHW (%)	53.8	32.9% (0.0–86.0)
Weight (kg)	93.5	99.3(91.5–101.7)
HbA1c (%)	5.9	5.9 (5.7–6.0)
FBG (mg/dl)	106.3	104.6 (99.0–107.6)
SBP (mm Hg)	124	128.7 (123.5–133.6)
DBP (mm Hg)	78	79.5 (77.2–82.5)
HDL (mg/dL)	46.0	46.1 (44.7–49.7)
Total Cholesterol (mg/dL)	NR	183.7 (183.0–192.1)
Screening method	Impaired fasting and elevated post-load glucose	Impaired fasting or elevated post load glucose or other high risk
Core lifestyle sessions offered	16	Mean 12.6 (Range 1–24, median 12.0)
Core lifestyle sessions attended	NR	Mean 11.0 (Range 3.67–25, median 10.1)
Type of sessions	100% Individual	16.7% individual, 70.8% group, 12.5% both
Setting	Clinical centers across US	Clinics, Community centers, Churches, Worksite, Internet
Duration of follow-up	Mean 2.8 y (Range 1–4 y)	Mean 9.3 mo, (Range 3–36, Median 12.0)
Maintenance	Yes (100%)	Sometimes (62.5%)
Delivery personnel	Mostly dieticians; also coaches with at least a master’s degree training in exercise physiology, behavioral psychology, or health education	Clinical personnel (physicians, nurses, dieticians, grad students) and nonclinical delivery (Internet-based classes and other lay community volunteers who underwent training)

NR = not reported; BMI = body mass index; HbA1c = hemoglobin A1c; NHW = non-Hispanic white; SBP = systolic blood pressure; DBP = diastolic blood pressure; FBG = fasting blood glucose; HDL = high density lipoprotein cholesterol

Programs amended the original DPP lifestyle intervention (Table 1) by changing the number or duration of core sessions offered (the DPP offered 16 in-person sessions over 24 wk), conducting group (instead of individual) sessions, modifying the type of lifestyle coach (DPP coaches were qualified dietitians, exercise physiologists, behavioral psychologists, or health educators), and changing or removing the monthly maintenance component where participants met or were contacted for the remainder of the follow-up period to promote continued adherence to healthy lifestyle principles.

The number of core sessions offered in included DPP-lifestyle programs ranged from 1 to 24, with a mean of 12.6 core sessions (median 12.0) offered and 11.0 mean core sessions attended (median 10.1). Thirty interventions incorporated a maintenance component, which varied from emails to intermittent in-person group sessions. Programs with scheduled maintenance ranged from 3–8 monthly sessions as follow-up after the initial core component. Mean study duration was 9.3 mo with a standard deviation (SD) of 5.5 mo and a range from 3 to 15 mo (median 12.0) Across all studies, overall attrition was 23.5% (range: 0.0 to 43.2).

### Outcomes

Across all studies, mean absolute pre-post weight change was -3.77 kg (95% CI: -4.55 to 2.99, I^2^ of 99.06% (95% CI: 98.96, 99.15) (Table 2). Aggregate data from 16 studies that randomly assigned participants to intervention or control groups showed 2.66 kg greater weight loss among intervention participants (-3.25 kg) compared to control participants (-0.59 kg). For further details, see forest plot in S13 Fig.

**Fig 2:**
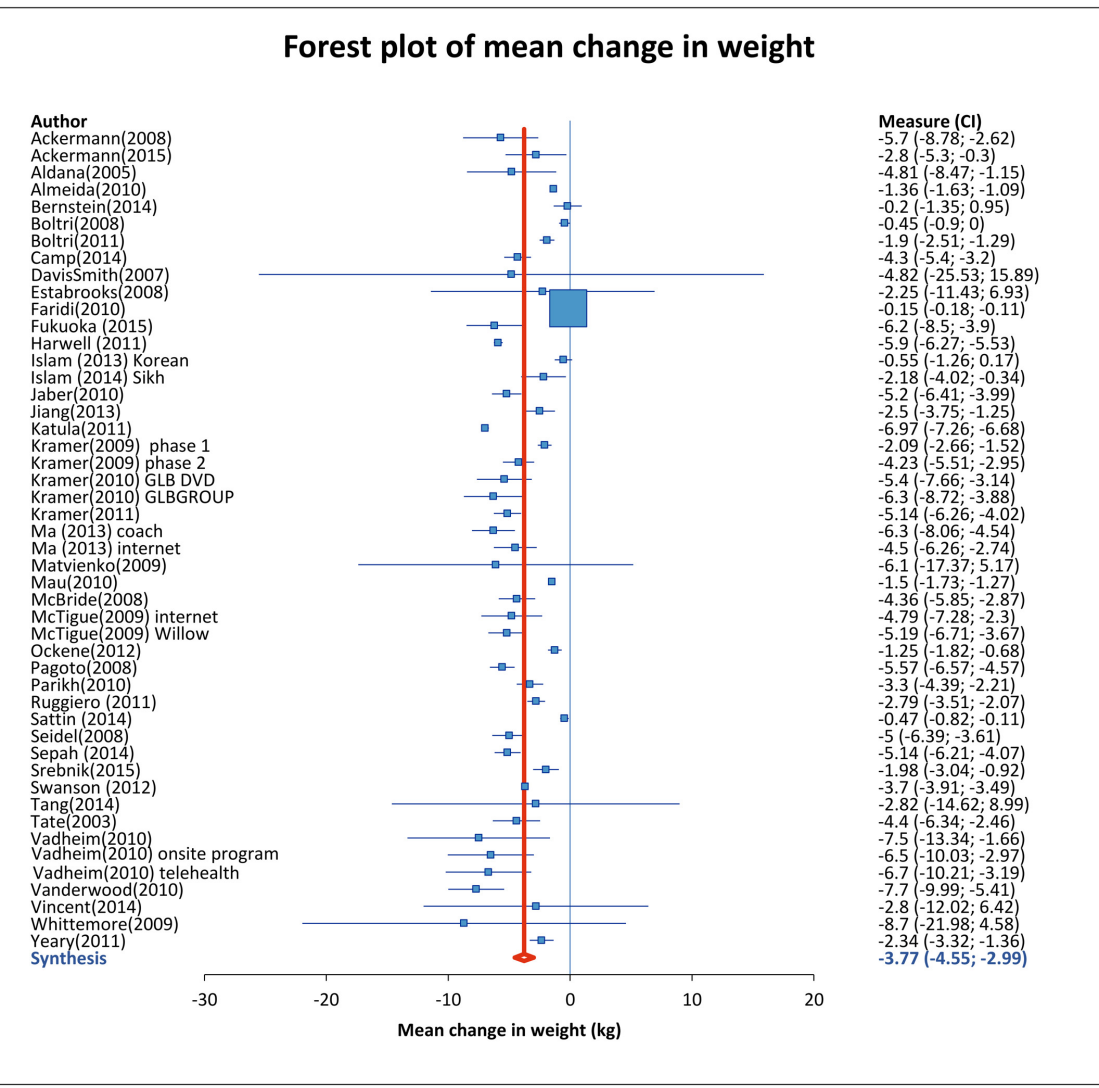
Mean weight change of study participants. Analysis of all 48 intervention groups which reported weight change from baseline to end of follow-up period. Data was analyzed with Mix 2.0 using random effects method with the weighting of each study proportional to the inverse of the variance. Study weight is indicated by the size of the box, and horizontal lines indicate the 95% confidence interval. The red line indicates the overall weight change, and summary diamond indicates pooled estimate with reported mean (95% CI).

**Table 2:** Comparison of outcomes from DPP versus Community Trials.

Value	Number of intervention groups	Mean* baseline	Mean change* at study completion (95% CI)	DPP trial at last DPP annual (avg 2.9 y)
Weight (kg)	48	99.3	-3.77 (-4.55, -2.99)	- 5.6
A1c (%)	8	5.9	-0.21 (-0.29, -0.13)	- 0.10
FBG (mg/dl)	21	104.6	-2.40 (-3.59, -1.21)	-5.0
SBP (mm Hg)	23	128.7	-4.29 (-5.73, -2.84)	-4.0
DBP (mm Hg)	22	79.5	-2.56 (-3.40, 1.71)	-4.0
HDL (mg/dL)	14	46.1	+0.85 (-0.10, 1.60)	+1.16
Total Cholesterol (mg/dL)	12	183.7	-5.34 (-9.72, -0.97)	NR

*Mean indicates average of study means

Among 8 studies that measured HbA1c, mean pre-post change was -0.21% (95% CI: -0.29; -0.13, I^2^ 82.72% [95% CI: 67.29%, 90.87.]) Among 21 studies reporting FBG, mean change in FBG was -2.40 mg/dl (95% CI: -3.59; -1.21, I^2^ 90.63% [95% CI: 87.08, 93.21]). Among the 23 studies evaluating BP, we observed a mean pre-post change in SBP of -4.29 mmHg (95% CI:-5.73, -2.84, I^2^ 75.40% [95% CI: 61.15, 84.43]) and -2.56 mmHg in DBP (95% CI:-3.40, 1.71, I^2^ 57.96% [95% CI: 29.09, 75.08]). There were 14 and 12 studies which reported on HDL and total cholesterol, respectively. There was an overall pre-post increase in HDL of +0.85 mg/dL (95% CI: -0.10, 1.60, I^2^ 63.12% [95% CI: 34.41, 79.26]) and change in TC of -5.34 mg/dl (95% CI: -9.72, -0.97, I^2^ 56.09% [95% CI: 16.19, 76.99]). There were insufficient data to perform between-group comparisons (intervention versus control) for outcomes other than weight.

### Stratified Analyses

Using our conservative definition of non overlapping CIs, studies with a maintenance component had a statistically significantly greater decrease in mean FBG (-3.14 mg/dl) and a greater decrease in weight (-1.66 kg) than intervention programs without a maintenance component (Table 3).

**Table 3:** Outcomes stratified by the presence of maintenance. (Supporting forest plot in S9 Fig.)

Outcome	Maintenance	95% CI	No maintenance	95% CI
Weight (kg)	-4.36	(-5.47,-3.26)	-2.70	(-3.59, -1.80)
FBG* (mg/dl)	-4.00	(-4.93, -3.07)	-0.86	(-2.75, 1.03)
SBP (mmHg)	-4.62	(-6.48, -2.76)	-3.81	(-6.34, -1.27)
DBP (mmHg)	-2.27	(-3.26, -1.29)	-3.34	(-5.08, -1.60)
HDL (mg/dl)	+1.31	(0.38, 2.23)	-0.22	(-1.45, 1.02)
TC (mg/dl)	-4.09	(-10.50, 2.32)	-6.33	(-15.00, -0.51)

* indicates statistically significant difference based on non-overlapping confidence intervals

No statistically or clinically significant differences in risk factor changes were observed when comparing studies testing interventions delivered by community workers to studies that employed health professionals, or those that used electronic media (Table 4). Similarly, no outcome differences were noted in studies classifying high-risk for diabetes based on blood glucose testing versus other criteria (Table 5), nor by study quality, (high versus average quality) or setting (clinic, community or remote). (Data shown in S7, S11 and S10 Figs).

**Table 4:** Outcomes stratified by type of provider for delivery of the intervention. (Supporting forest plot in S8 Fig.)

Outcome	Electronic	(95% CI)	Community	(95% CI)	Clinical	(95% CI)
Weight (kg)	-5.02	(-5.72, -4.32)	-3.13	(-4.66, -1.59)	-3.77	(-4.66, -2.88)
FBG (mg/dl)	-3.08	(-5.22, -0.94)	+1.78	(-4.47, 8.04)	-2.87	(-4.34, -1.40)
SBP (mmHg)	-4.26	(-7.21, -1.30)	-4.06	(-6.84, -1.28)	-4.53	(-6.80, -2.27)
DBP (mmHg)	-1.34	(-3.24, 0.56)	-2.64	(-4.29, -1.00)	-2.97	(-4.14, -1.80)
HDL (mg/dl)	+0.64	(-1.22, 2.51)	+1.92	(-0.92, 4.75)	+0.87	(-0.05, 1.79)
TC (mg/dl)	-5.76	(-15.3, 3.77)	-0.63	(-24.47, 25.74)	-5.80	(-10.48, -1.12)

**Table 5:** Outcomes stratified by method used to determine “high risk” status. (Supporting forest plots in S7 Fig.)

Outcome	Blood glucose criteria	(95% CI)	Risk factor criteria	(95% CI)	Either	(95% CI)
Weight (kg)	-3.23	(-4.52, -1.95)	-3.62	(-4.67, -2.57)	-5.04	(-6.34, -3.74)
FBG (mg/dl)	-2.40	(-4.12, -0.67)	-0.66	(-5.17, 3.84)	-3.27	(-4.62, -1.92)
SBP (mmHg)	-2.46	(-4.07; -0.84)	-4.92	(-8.12, -1.72)	-5.04	(-7.46, -2.62)
DBP (mmHg)	-4.66	(-7.54; -1.77)	-1.91	(-3.40, -0.42)	-2.74	(-3.52, -1.96)
HDL (mg/dl)	+0.46	(-0.47, 1.39)	+0.99	(-0.95, 2.92)	+1.13	(-0.31, 2.57)
TC (mg/dl)	-11.94	(-17.80, -6.08)	+0.57	(-10.83, 22.23)	-5.47	-(10.48,-0.46)

## Discussion

This is the first meta-analysis to aggregate both weight and cardiovascular risk factor changes from US community-based studies of DPP-based lifestyle interventions. Characteristics of participants in these studies of DPP lifestyle programs were very similar to those of the original trial participants, but translation study participants had a slightly higher mean starting weight and higher proportion of females [13]. The original DPP participants had a greater mean weight loss at 1 y than the participants in this meta-analysis (6.8 kg versus 3.8 kg), which was likely due to the more resource intensive intervention and individualized support in the trial [13]. However, this weight change was closer to the 4.2 kg weight loss reported in the Finnish Diabetes Prevention Study (DPS) [11]. Studies with adequate control groups showed an additional 1.9 kg weight lost across intervention arms when compared with their respective control arms (3.3 versus 0.6 kg). Indeed, the control groups in these effectiveness studies achieved some benefit from participation, even if only exposed to minimal intervention. Compared to the original DPP, HbA1c, and SBP reductions observed in translation studies were similar; FBG and DBP reductions were somewhat lower than the reductions achieved in the efficacy trial; and comparisons for HDL and TC were not possible.

We noted no difference in cardiometabolic risk factor changes in people with biochemically confirmed prediabetes versus those with diabetes risk factors. That said, progression to diabetes and its complications varies by type of prediabetes. The DPP enrolled patients with both IGT and IFG, who are at approximately three times higher risk of progression to diabetes compared to those with IFG alone [7]. The Finnish DPS, Malmo, and Da Qing studies also included participants with IGT or combined IGT and IFG, who are at higher risk than those with IFG alone [7,9,14]. Meanwhile, the US-based DPP-translation studies primarily used IFG criteria, and none used oral glucose tolerance tests to determine high-risk status. This suggests that participants in these studies had a lower risk profile, which was also reflected in their lower baseline FBG and HbA1c levels. Assuming the participants in this analysis were at a lower baseline risk, the changes in cardiometabolic risks observed were commensurate with the starting risk level, and are therefore still noteworthy. It remains unclear whether the DPP intervention is effective in preventing diabetes among participants with impaired fasting glucoses but normal post-load glucose levels. Also, given ease of testing, HbA1c is now commonly used to diagnose prediabetes, and it is unclear whether DPP results can be extended to this prediabetes population defined by HbA1c.

Our findings are also similar to other recent studies. A Community Guide Review, which evaluated interventions across diverse countries and settings, had similar decreases in FPG with a nonsignificant trend towards decreased blood pressure and cholesterol [22]. The MOVE! program evaluated a ten-module program among 238,000 veterans; high intensity intervention participants achieved 2.7% weight loss at 6 mo compared to a 0.6% weight loss in the low intensity group [13,74]. Our findings were also similar to a systematic review and meta-analysis that pooled 22 studies published before July 2012 that translated diabetes prevention for real-life settings in multiple countries (US, Australia, Europe, and Japan) and had ≥12 mo of follow-up [75]. Since multiple countries were involved, heterogeneous study interventions were benchmarked to Europe-wide diabetes prevention implementation guidelines and showed overall pre-post changes in weight (-2.32 kg), HbA1c (-0.13 mmol/mol), FPG (-0.10 mmol/L [-1.8 mg/dl]), SBP and DBP (-4.30/-4.28 mmHg), HDL (+0.01 mmol/L [+0.39 mg/dl]), and TC (-0.18 mmol/L [-6.96 mg/dl]); importantly, greater adherence to recommendations was associated with larger weight reduction. Our study expands on this work with a larger number of pooled studies and participants, all using a similar core set of intervention principles and comparison to the original DPP Study.

Effective translation of a program depends on multiple components, including referral, uptake, engagement, completion, and post-program sustainability of outcomes in the whole population. In our review, after eligibility criteria was applied, 25.5% of all eligible participants did not enroll; of those who enrolled, there was an additional 23.8% attrition. Rates of attrition also inherently select for those who are the most motivated participants, which biases the results towards effectiveness. This limits the generalizability of our findings, which more accurately apply to those who complete the program.

Implementation of DPP lifestyle programs have been limited by both cost and sustainability of ongoing program participation and risk factor reductions [76]. Most of the programs studied in this analysis provided free testing and intervention supplies but offered few additional incentives to encourage participation. The most common methods used to decrease cost were modifications to the intervention, such as offering the intervention in accessible locations, delivering the intervention through lay providers, and taking advantage of group classes and electronic delivery options. Over 80% of studies tested group interventions, most had fewer mean core sessions compared to DPP, and only 60% offered a maintenance component. Importantly, we noted similar risk factor benefits were achievable in interventions delivered by different providers in both group and individual formats. The similarities in weight loss and secondary outcomes compared to the DPP is encouraging for the ability to make the intervention cost-effective without sacrificing the effectiveness. With options that include group sessions, community-based programs with social support, cultural tailoring, and remote low-cost maintenance such as text messages or phone calls, the interventions allow for scaling to a wider audience.

Reach and sustainability of behavior change interventions remain, as do other challenges of implementing diabetes prevention. The advantages of community-based interventions that were pooled in this study include familiar context, peer support, and convenience to facilitate continued participation. The success of electronic and remote interventions is also encouraging, as these could be distributed nationally with ease. The option of pre-recorded workouts on in-home cable TV illustrates a low-cost method of delivery that does not necessitate travel and is available on demand, in contrast to on-site workout regimens for which participants pay to participate [77]. This preliminary analysis also suggests that programs that implemented a maintenance component after the completion of the core sessions had greater reductions in weight and fasting glucose. The duration and intensity of maintenance that is most effective and the utility after the 1 y mark is largely unknown. Further evaluation of types of maintenance programs following a year-long program would be helpful to understand long-term benefit and sustainability.

### Limitations

A key limitation of our analysis was the heterogeneity of the studies included, which is inherent in all meta-analyses. Differences in duration of follow-up (from 1.5 to 36 mo), location of delivery, and other delivery format adaptations of the original DPP program were the most likely sources of heterogeneity. However, as the intervention (DPP-lifestyle program principles) and outcomes were similar, this study adds to the literature by providing external validity and noteworthy pre-post and between group cardiometabolic risk factor changes.

The lack of statistical significance found in most of the stratified analyses is likely due to lack of statistical power, which resulted in large, overlapping confidence intervals, as well as our conservative definition of statistical significance based only on non-overlapping CI’s. As most meta-analyses, our study is confined to the use of previously reported results. We used a more conservative definition of statistical significance by comparing stratum specific results, though this did not allow a more consistent adjustment for confounders. However, a less conservative analytical approach may have found other program characteristics that had “statistically significant” associations with cardiometabolic changes.

Additionally, studies varied significantly in quality. We conducted a sensitivity analysis to evaluate change in weight stratified by study quality (high versus average quality), which showed no significant difference. However, this alone is not expected to account for variation that arises during recruitment, enrollment, and study conduct. The majority of studies in our review used pre-post single group study designs and may be subject to confounding. To address this, we separately examined studies that had control groups and demonstrated that intervention groups achieved larger benefits than control groups.

### Conclusion

Delivery of lifestyle programs adhering to DPP principles tested in community and clinical settings achieved similar 1 y decreases in weight, FBG, and HbA1c as the original DPP study, despite the modifications made to lower cost and improve acceptability across various settings. Though unclear if these changes truly translate into reductions in diabetes incidence, prior studies have found decreased incidence to be most closely related to weight loss [13]. Methods to increase uptake and decrease attrition are both needed to enable long-lasting, sustainable lifestyle change in patients with the highest risk of progression to diabetes and its associated complications.

### Disclaimer

The findings and conclusions in this report are those of the authors and do not necessarily represent the official position of the US Centers for Disease Control and Prevention.

## Supporting Information

S1 PRISMA Checklist This checklist (preferred reporting items for systematic reviews and meta-analysis protocols) indicates the location of each item that is the part of the recommended set of items to report in systematic reviews and meta-analyses.
(DOC)

S1 Fig Forest plot of FBG. Forest plot of all studies that evaluated change in FPG, with I^2^ of 90.63% (95% CI: 87.08, 93.21).
(TIFF)

S2 Fig Forest plot of HbA1c. Forest plot of all studies that evaluated change in A1c, with I^2^ of 82.72% (95% CI: 67.29%, 90.87).
(TIFF)

S3 Fig Forest plot of SBP. Forest plot of all studies that evaluated change in SBP, with I^2^ of 75.40% (95% CI: 61.15, 84.43).
(TIFF)

S4 Fig Forest plot of DBP. Forest plot of all studies that evaluated change in DBP, with ^I2^ of 57.96% (95% CI: 29.09, 75.08).
(TIFF)

S5 Fig Forest plot of HDL. Forest plot of all studies that evaluated change in HDL, with I^2^ of 63.12% (95% CI: 34.41, 79.26).
(TIFF)

S6 Fig Forest plot of TC. Forest plot of all studies that evaluated change in TC, with I^2^ of 56.09% (95% CI: 16.19, 76.99).
(TIFF)

S7 Fig Forest plot of weight change stratified by method of risk classification. Forest plot of weight change stratified by method used to determine high risk. Listed first are studies that used blood glucose testing or previously defined prediabetes. Listed second are studies that defined high risk by BMI with one additional risk factor. Listed last are studies that allowed participation if either of the above criteria was met.
(TIFF)

S8 Fig Forest plot of weight change stratified by type of provider. Forest plot of weight change stratified by type of provider who delivered the intervention. Listed first are studies that had an electronic method of delivery (DVD, media, internet), second are those that used a lay community member who had been trained to deliver the intervention, and third are providers with a health care background.
(TIFF)

S9 Fig Forest plot of weight change stratified by maintenance. Forest plot of weight change stratified by the presence of maintenance. Listed first are studies that had only core sessions, and second are studies that had a component of maintenance after the core intervention.
(TIFF)

S10 Fig Forest plot of weight change stratified by quality. Forest plot of weight change stratified by quality. Listed first are studies that had fewer than two indicators of quality (see original manuscript) and second are those that had at least two indicators of quality.
(TIFF)

S11 Fig Forest plot of weight change stratified by location. Forest plot of weight change stratified by the location of the intervention. Listed first are studies that were conducted remotely (such as in participants’ home), second are those conducted in a community setting (such as a worksite or church), and third are those based out of clinics.
(TIFF)

S12 Fig Forest plot of weight change stratified by type of session. Forest plot of weight change stratified by type of class in the intervention. Listed first are those that used a combination of group and individual classes, second are those that had group classes, and last are those that had individual classes.
(TIFF)

S13 Fig Forest plot of weight change in all studies with control group data. Forest plot of weight change in all studies that reported data for participants in an intervention and a control arm. Listed first are data from the control group participants, and second are data from their respective studies with a duration of 12 mo or greater.
(TIFF)

S1 Flow Diagram This flow diagram includes all the records that were identified at each stage of the search with the PRISMA Flow Diagram template. After the application of inclusion and exclusion criteria, 44 studies met criteria and were included in the final analysis.
(DOC)

S1 Table List of search terms. (DOCX)

S2 Table Baseline participant and intervention characteristics by study. (DOCX)

S3 Table Scoring system adapted from the Juni scoring criteria. (DOCX)

## Abbreviations

BMI: body mass index
DBP: diastolic blood pressure
DPP: Diabetes Prevention Program
DPS: Diabetes Prevention Study
FBG: fasting blood glucose
GDM: gestational diabetes
HbA1c: glycosylated hemoglobin
HDL: high density lipoprotein
IFG: impaired fasting glucose
IGT: impaired glucose tolerance
NHW: non-Hispanic white
SBP: systolic blood pressure
TC: total cholesterol
