# Supplementary figures and images for: Cardiometabolic Risk Factor Changes Observed in Diabetes Prevention Programs in US Settings: A Systematic Review and Meta-analysis
Source: PLoS Med. 2016 Jul 26;13(7):e1002095. doi: 10.1371/journal.pmed.1002095 (PMC4961455; doi:10.1371/journal.pmed.1002095)

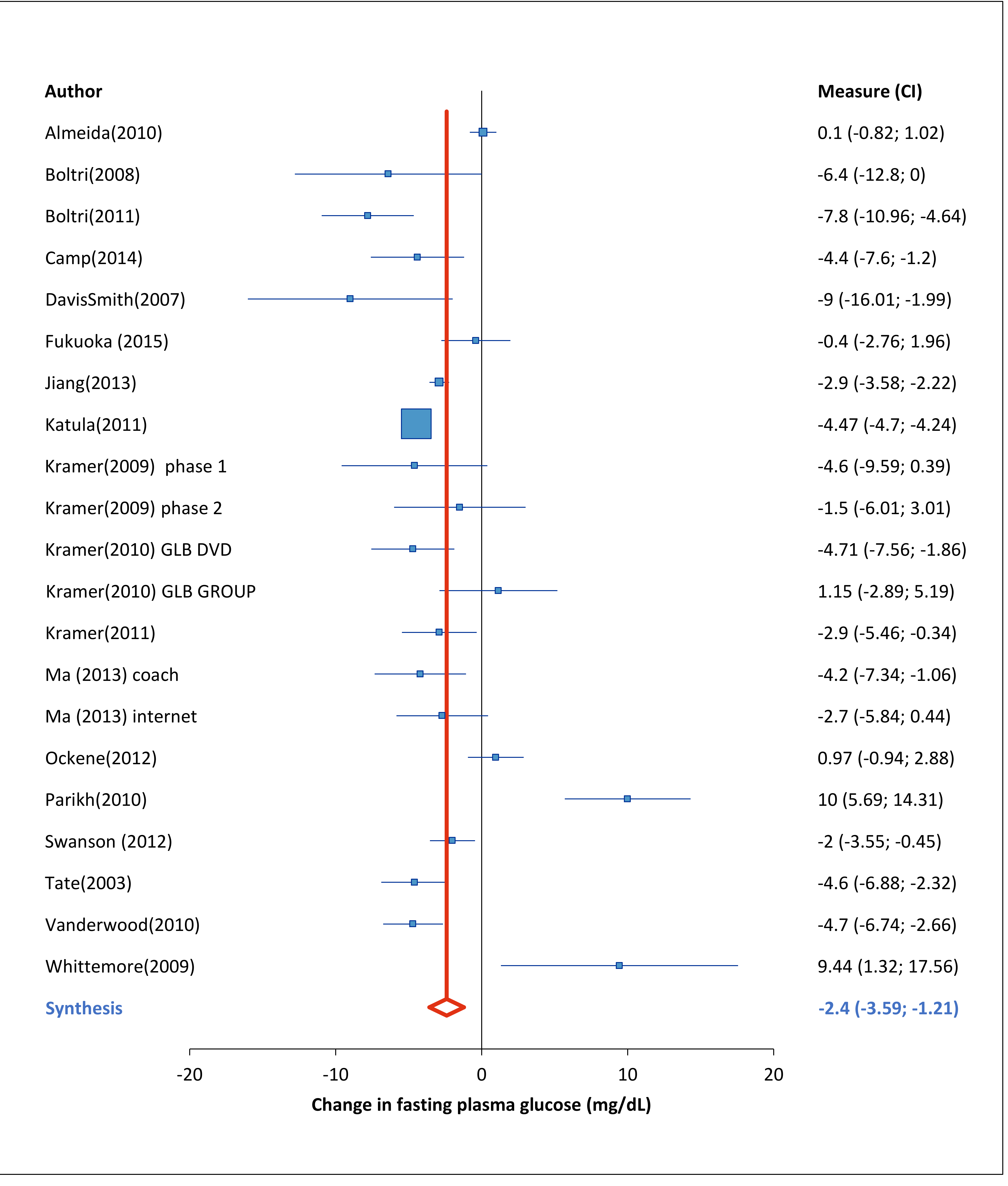

Supplement: S1 Fig — Forest plot of all studies that evaluated change in FPG, with I2 of 90.63% (95% CI: 87.08, 93.21). (TIFF) [file pmed.1002095.s002.tiff]

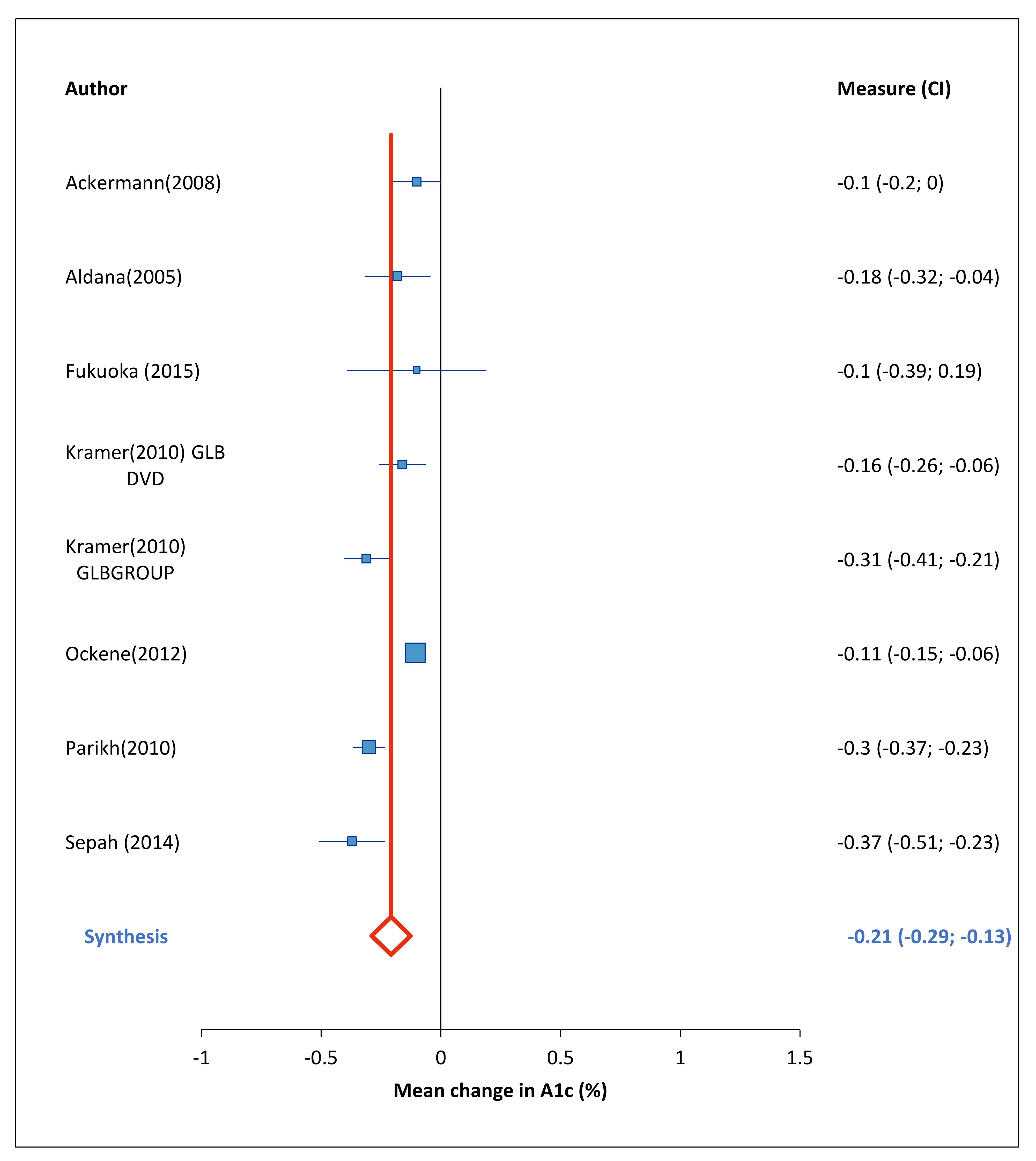

Supplement: S2 Fig — Forest plot of all studies that evaluated change in A1c, with I2 of 82.72% (95% CI: 67.29%, 90.87). (TIFF) [file pmed.1002095.s003.tiff]

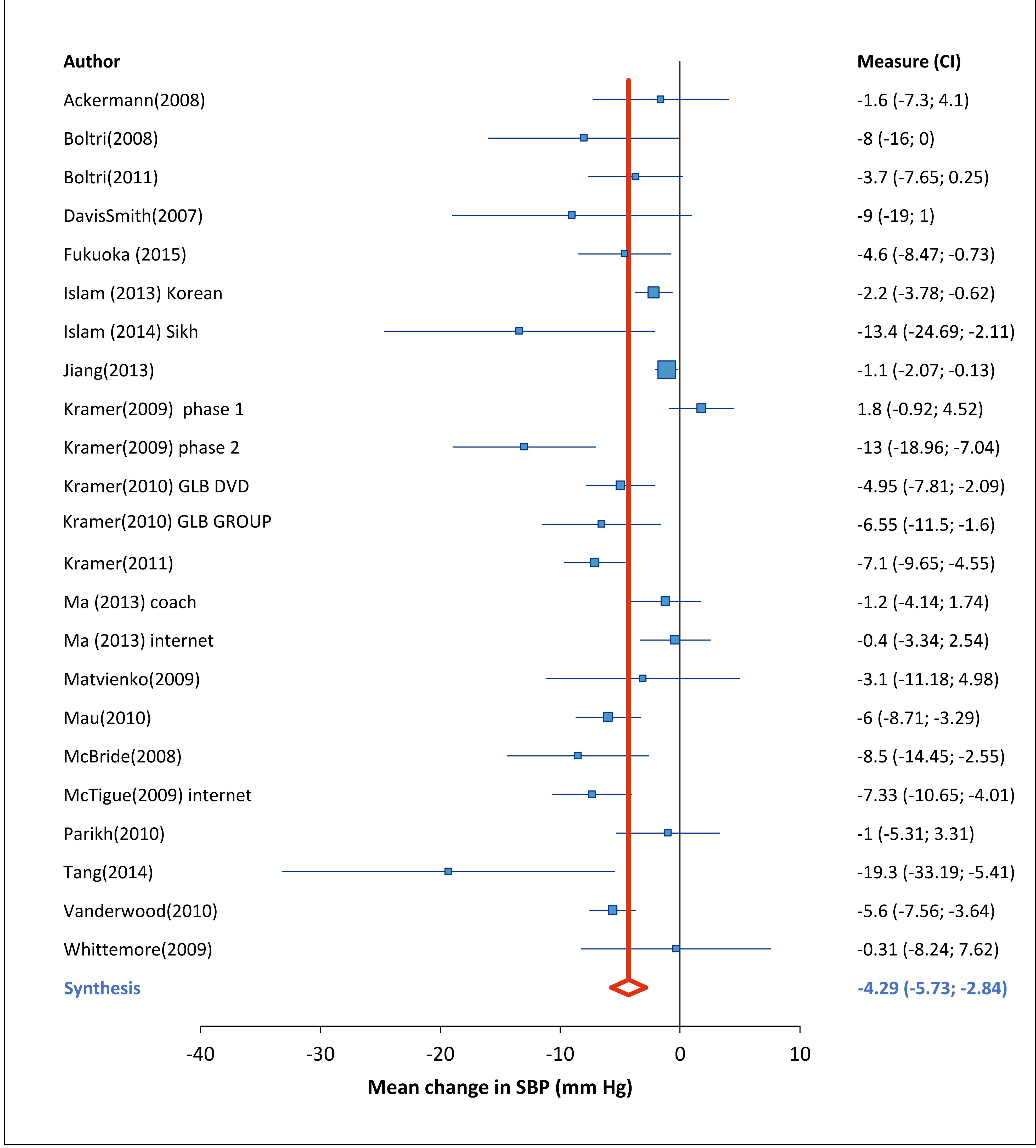

Supplement: S3 Fig — Forest plot of all studies that evaluated change in SBP, with I2 of 75.40% (95% CI: 61.15, 84.43). (TIFF) [file pmed.1002095.s004.tiff]

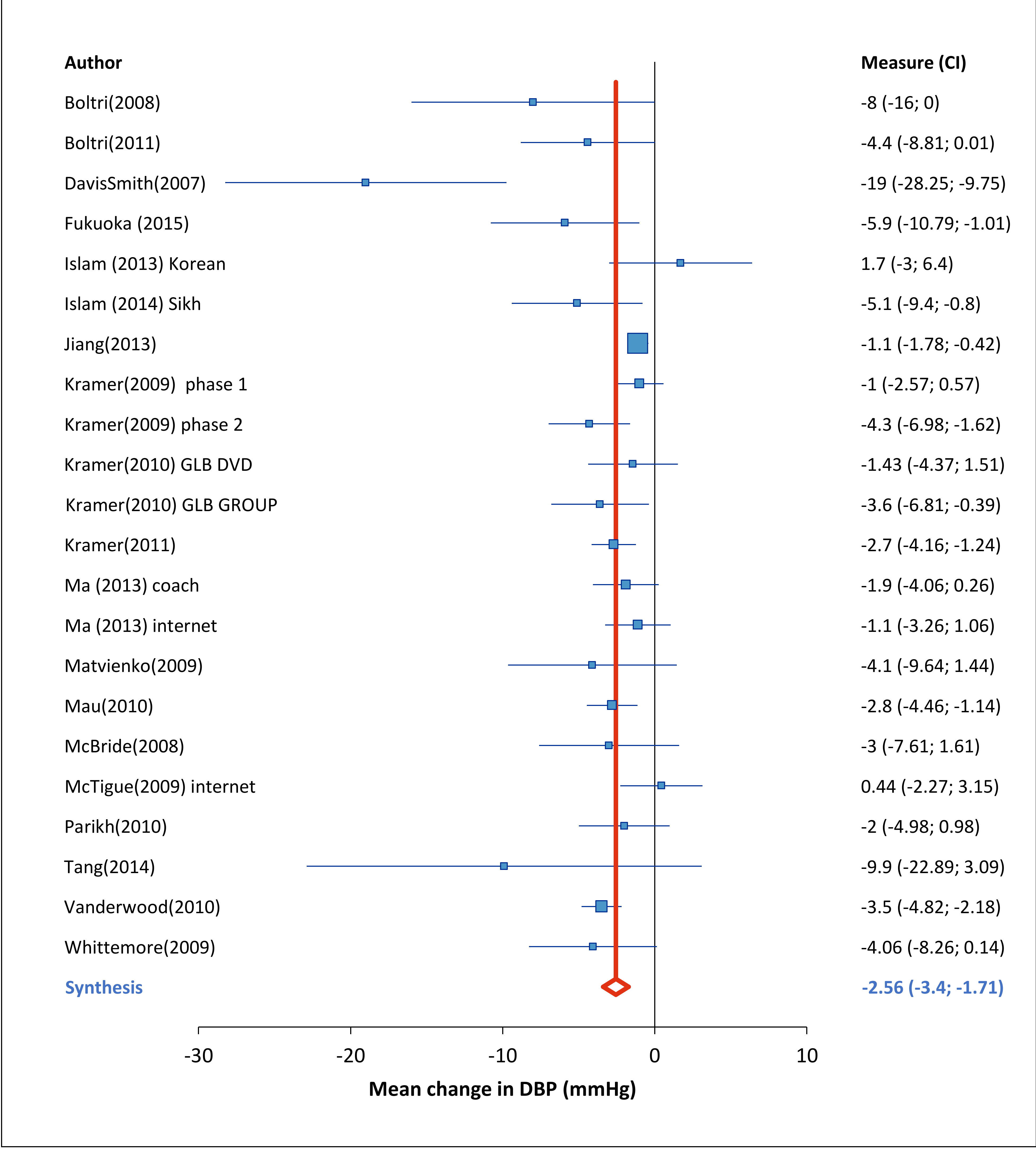

Supplement: S4 Fig — Forest plot of all studies that evaluated change in DBP, with I2 of 57.96% (95% CI: 29.09, 75.08). (TIFF) [file pmed.1002095.s005.tiff]

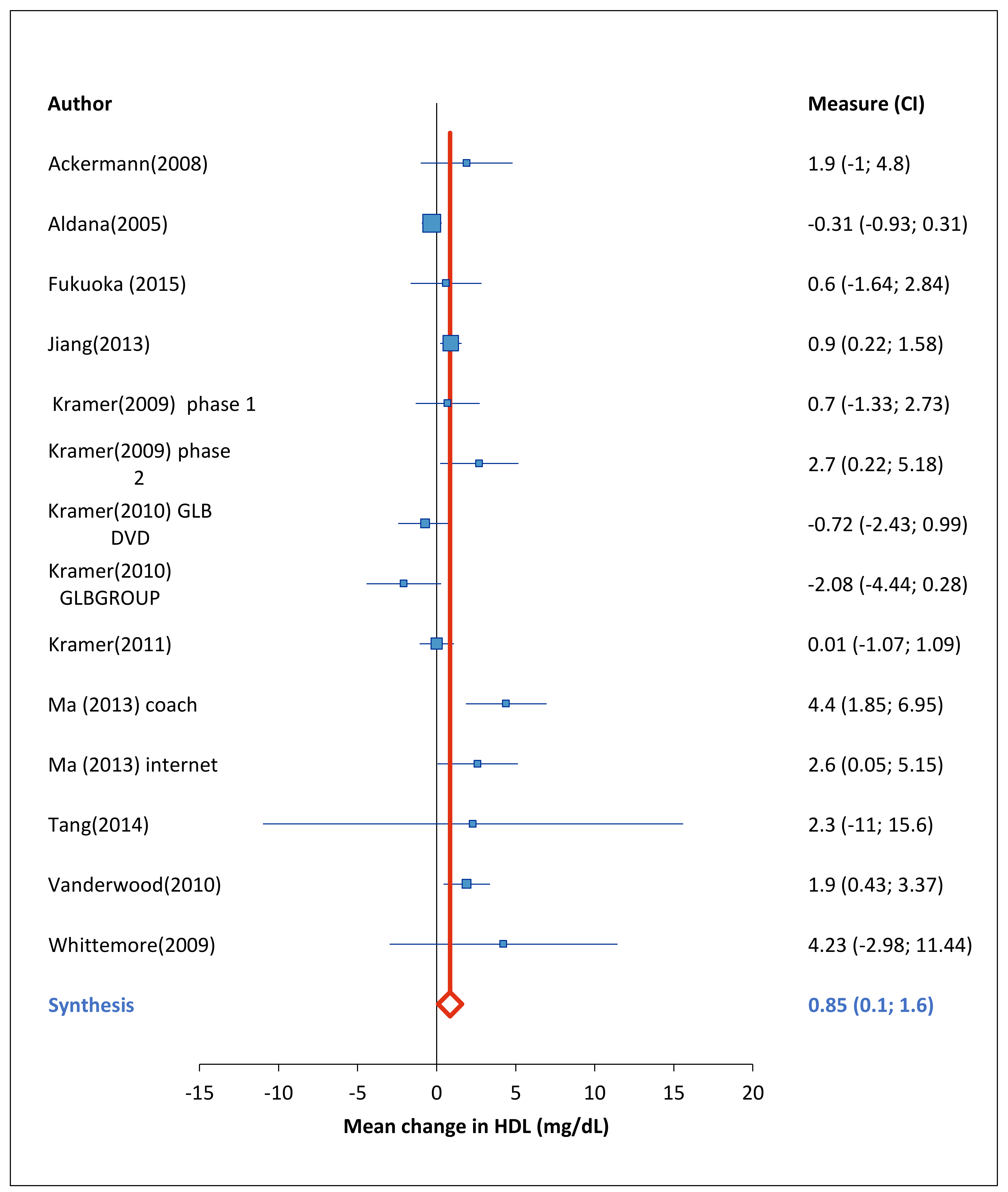

Supplement: S5 Fig — Forest plot of all studies that evaluated change in HDL, with I2 of 63.12% (95% CI: 34.41, 79.26). (TIFF) [file pmed.1002095.s006.tiff]

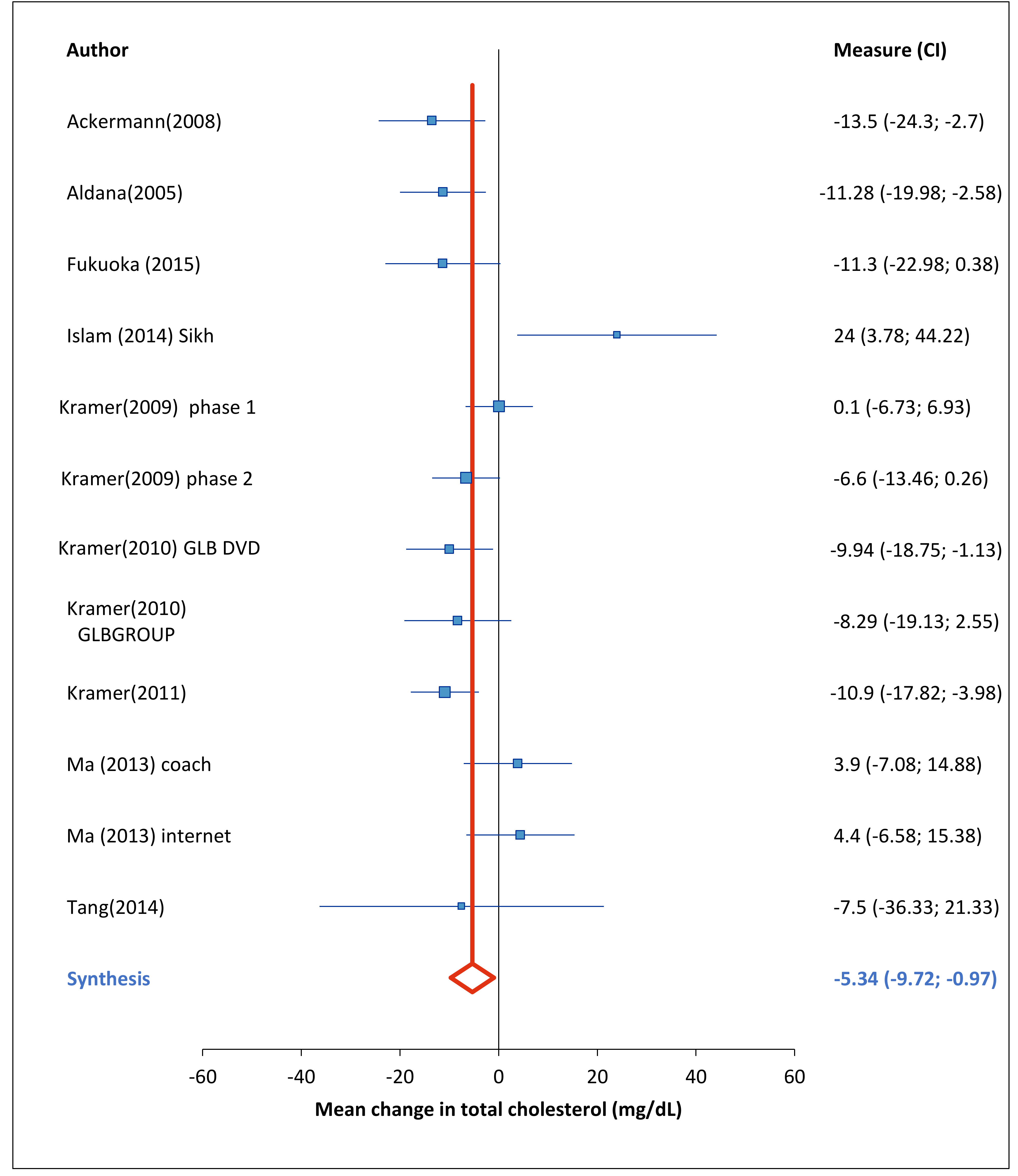

Supplement: S6 Fig — Forest plot of all studies that evaluated change in TC, with I2 of 56.09% (95% CI: 16.19, 76.99). (TIFF) [file pmed.1002095.s007.tiff]

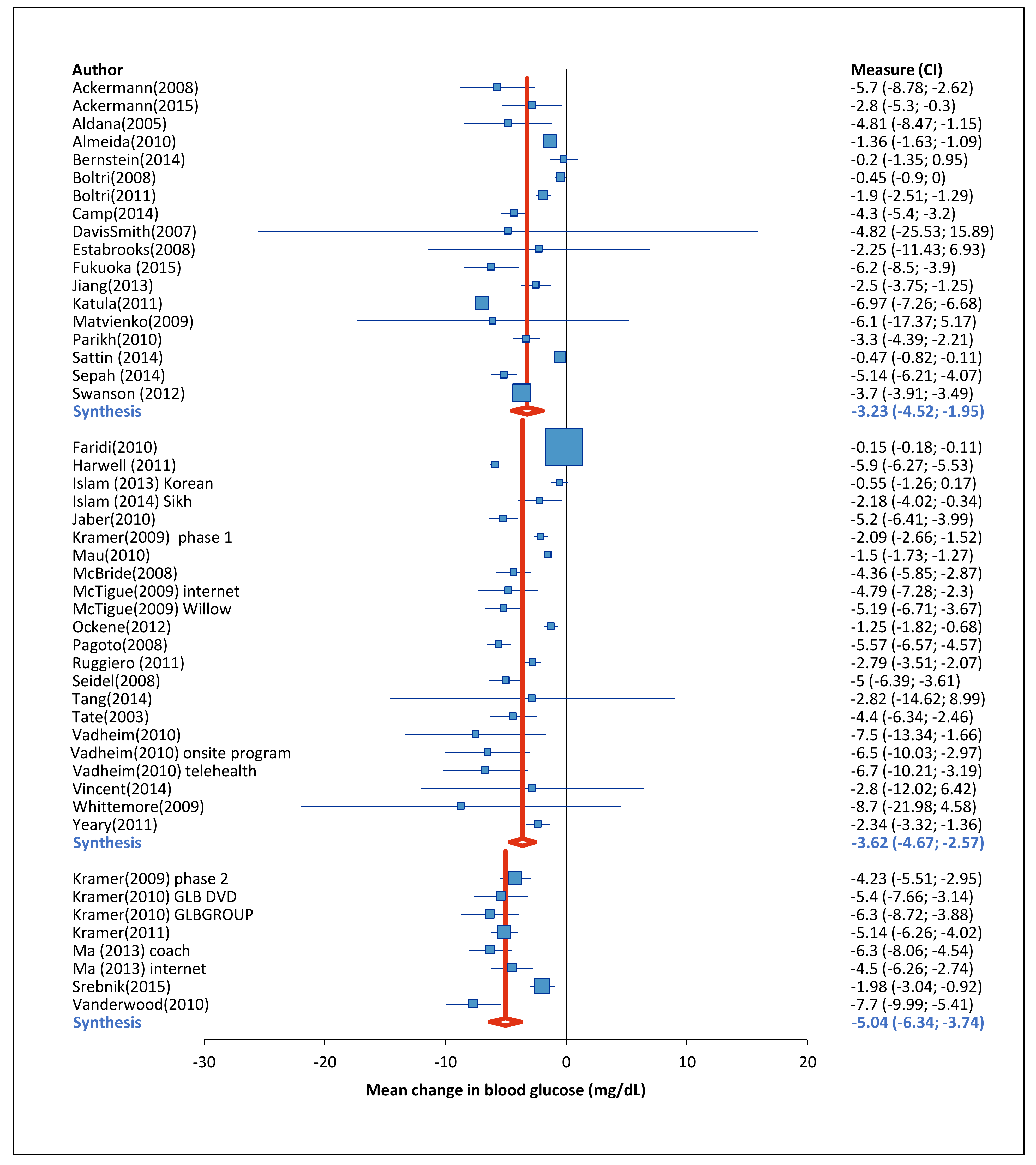

Supplement: S7 Fig — Forest plot of weight change stratified by method used to determine high risk. Listed first are studies that used blood glucose testing or previously defined prediabetes. Listed second are studies that defined high risk by BMI with one additional risk factor. Listed last are studies that allowed participation if either of the above criteria was met. (TIFF) [file pmed.1002095.s008.tiff]

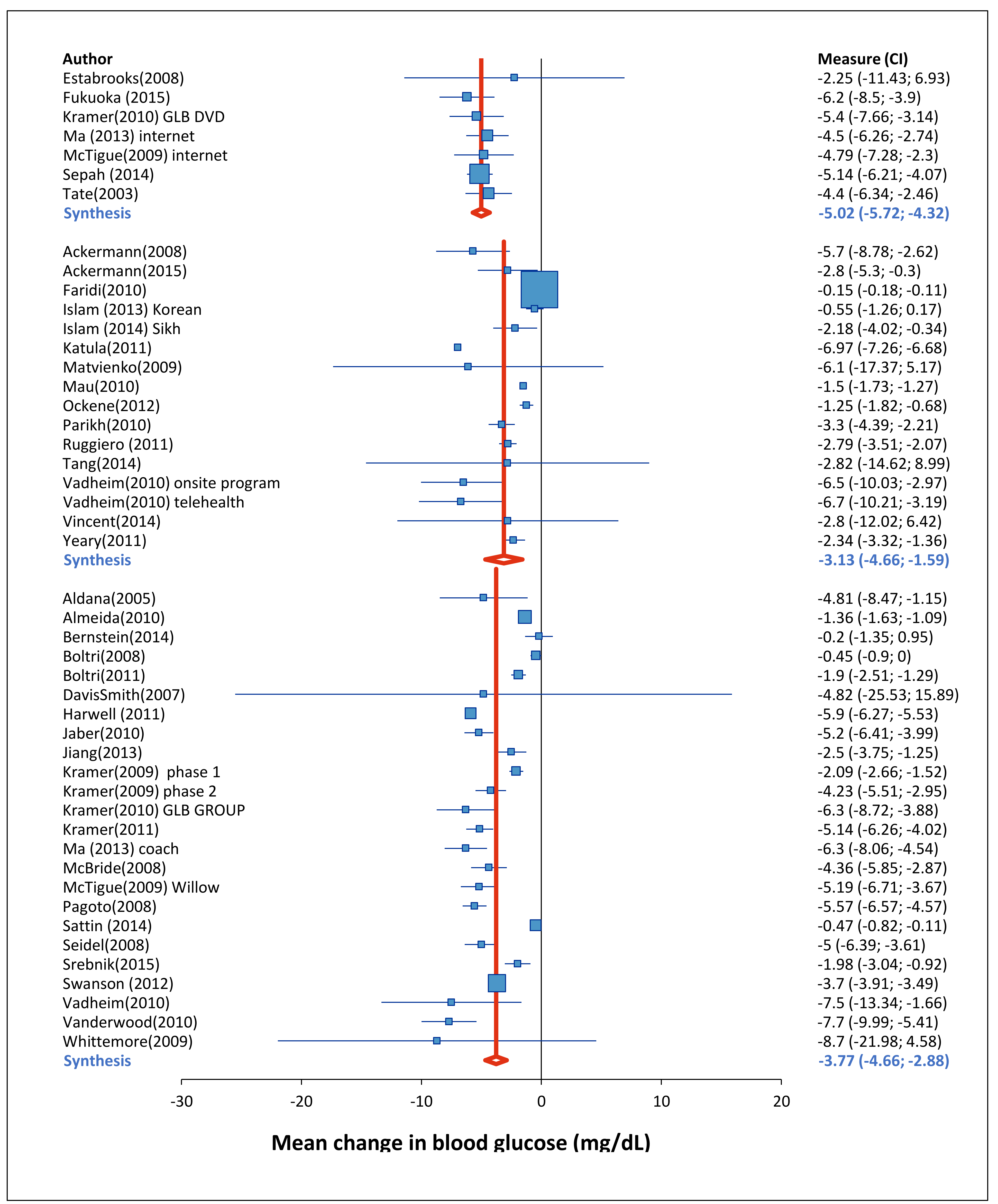

Supplement: S8 Fig — Forest plot of weight change stratified by type of provider who delivered the intervention. Listed first are studies that had an electronic method of delivery (DVD, media, internet), second are those that used a lay community member who had been trained to deliver the intervention, and third are providers with a health care background. (TIFF) [file pmed.1002095.s009.tiff]

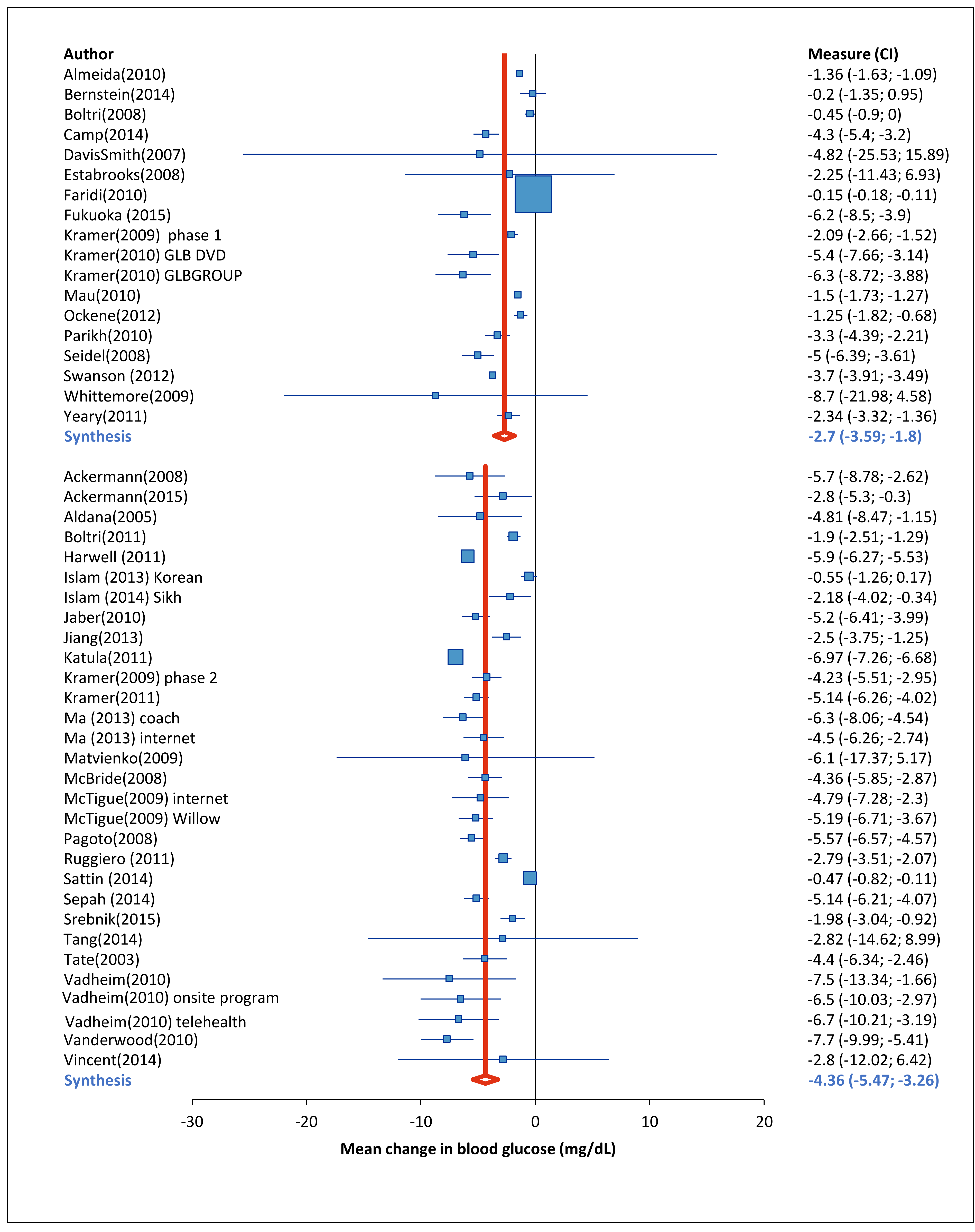

Supplement: S9 Fig — Forest plot of weight change stratified by the presence of maintenance. Listed first are studies that had only core sessions, and second are studies that had a component of maintenance after the core intervention. (TIFF) [file pmed.1002095.s010.tiff]

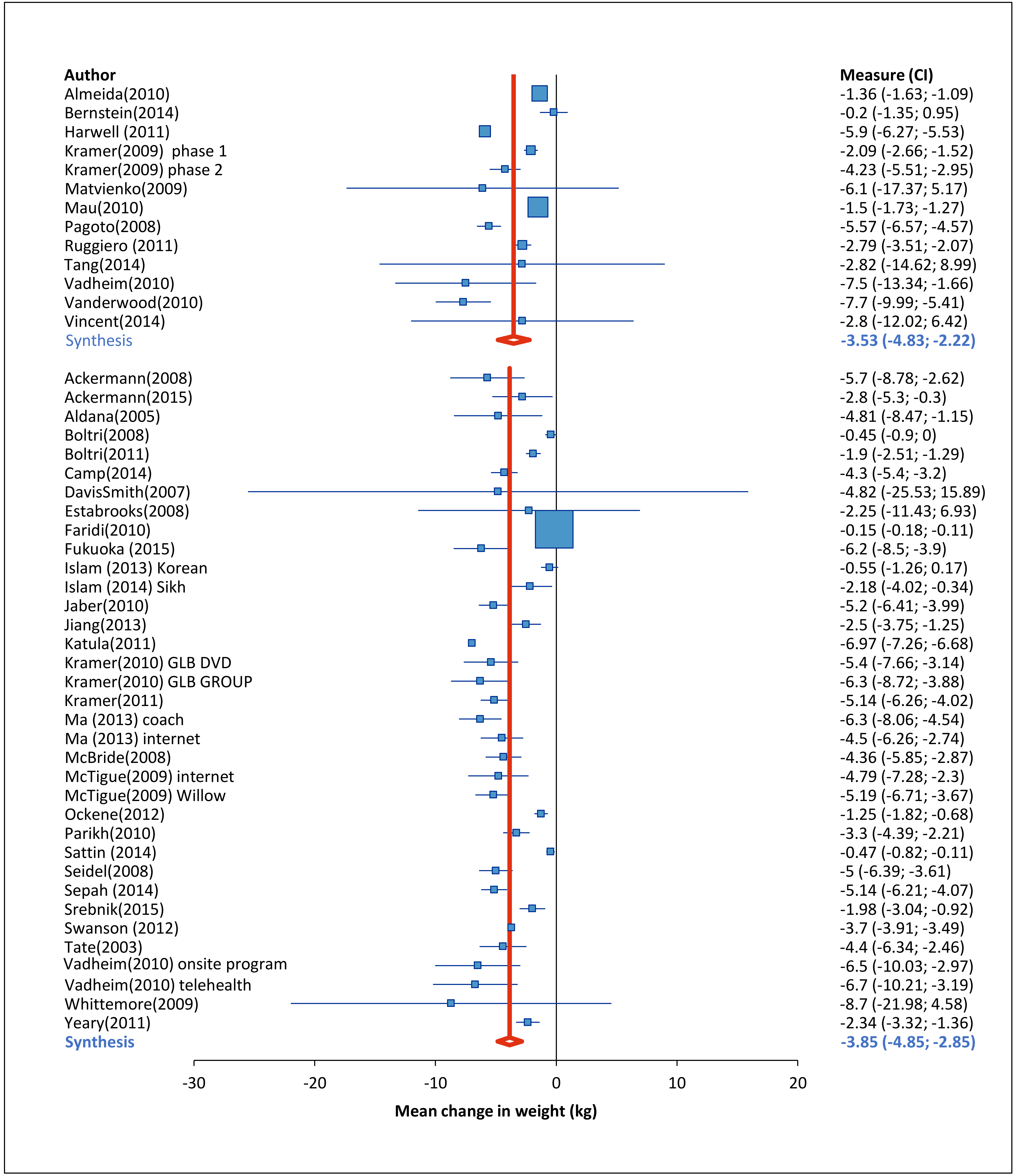

Supplement: S10 Fig — Forest plot of weight change stratified by quality. Listed first are studies that had fewer than two indicators of quality (see original manuscript) and second are those that had at least two indicators of quality. (TIFF) [file pmed.1002095.s011.tiff]

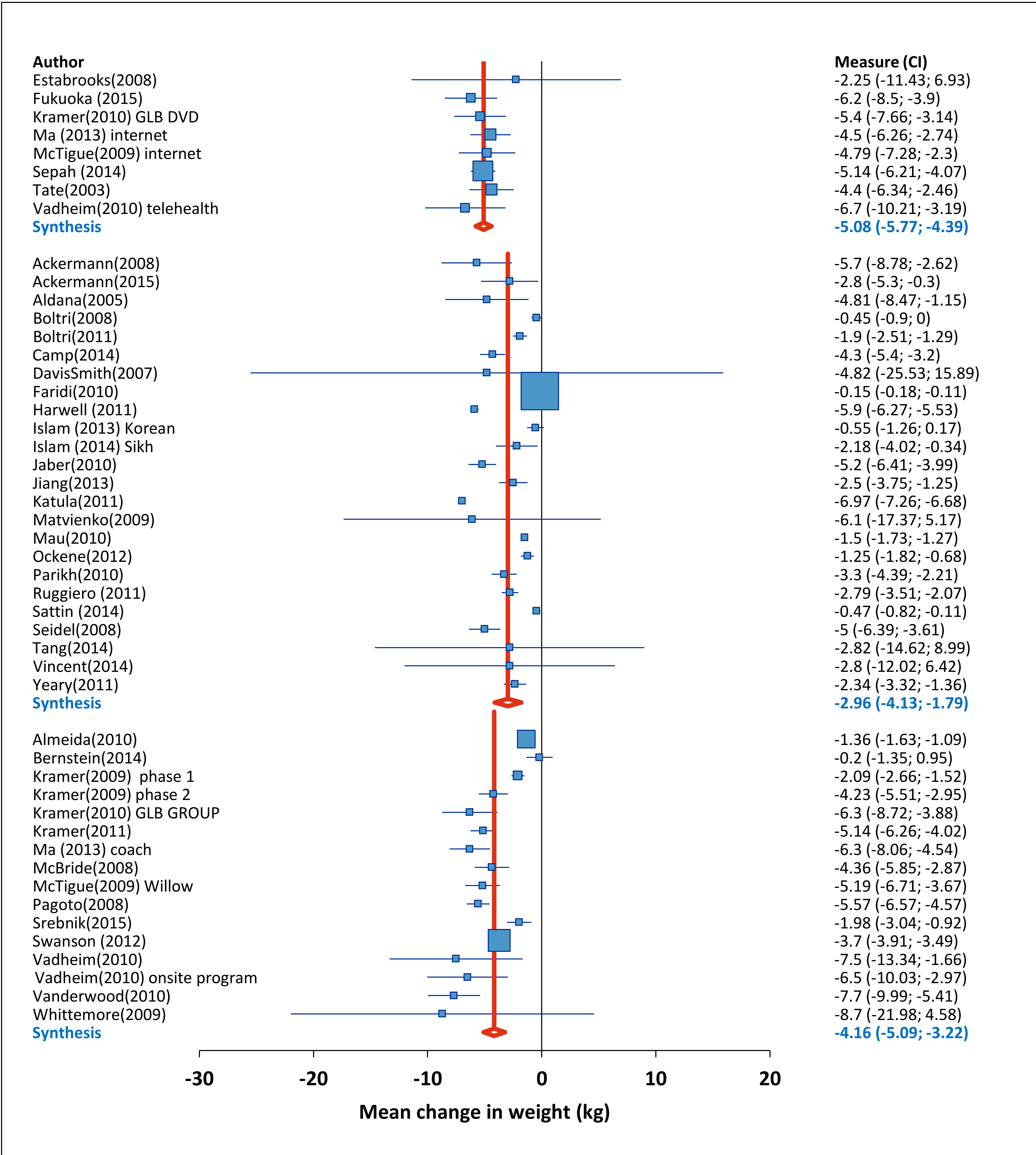

Supplement: S11 Fig — Forest plot of weight change stratified by the location of the intervention. Listed first are studies that were conducted remotely (such as in participants’ home), second are those conducted in a community setting (such as a worksite or church), and third are those based out of clinics. (TIFF) [file pmed.1002095.s012.tiff]

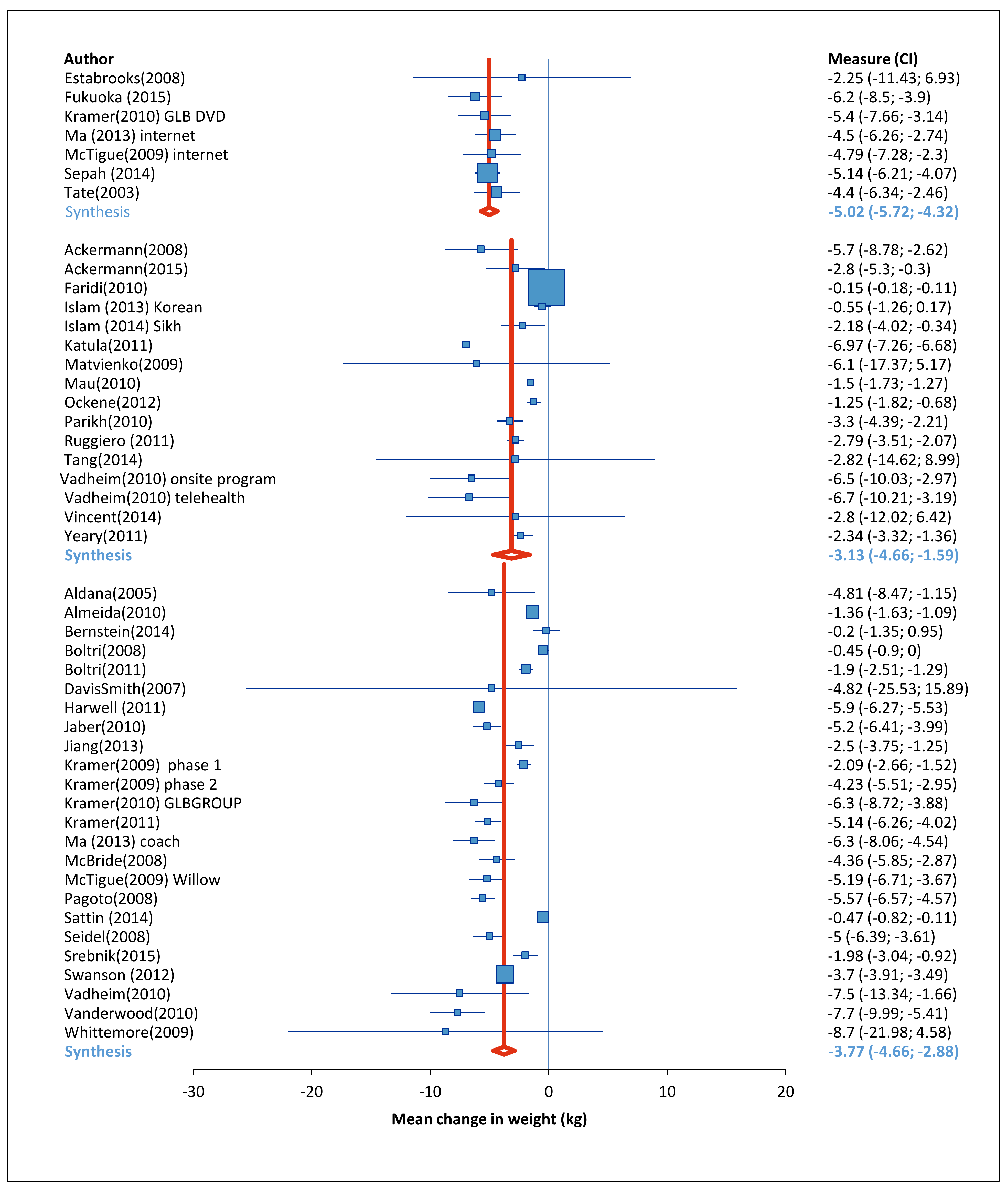

Supplement: S12 Fig — Forest plot of weight change stratified by type of class in the intervention. Listed first are those that used a combination of group and individual classes, second are those that had group classes, and last are those that had individual classes. (TIFF) [file pmed.1002095.s013.tiff]

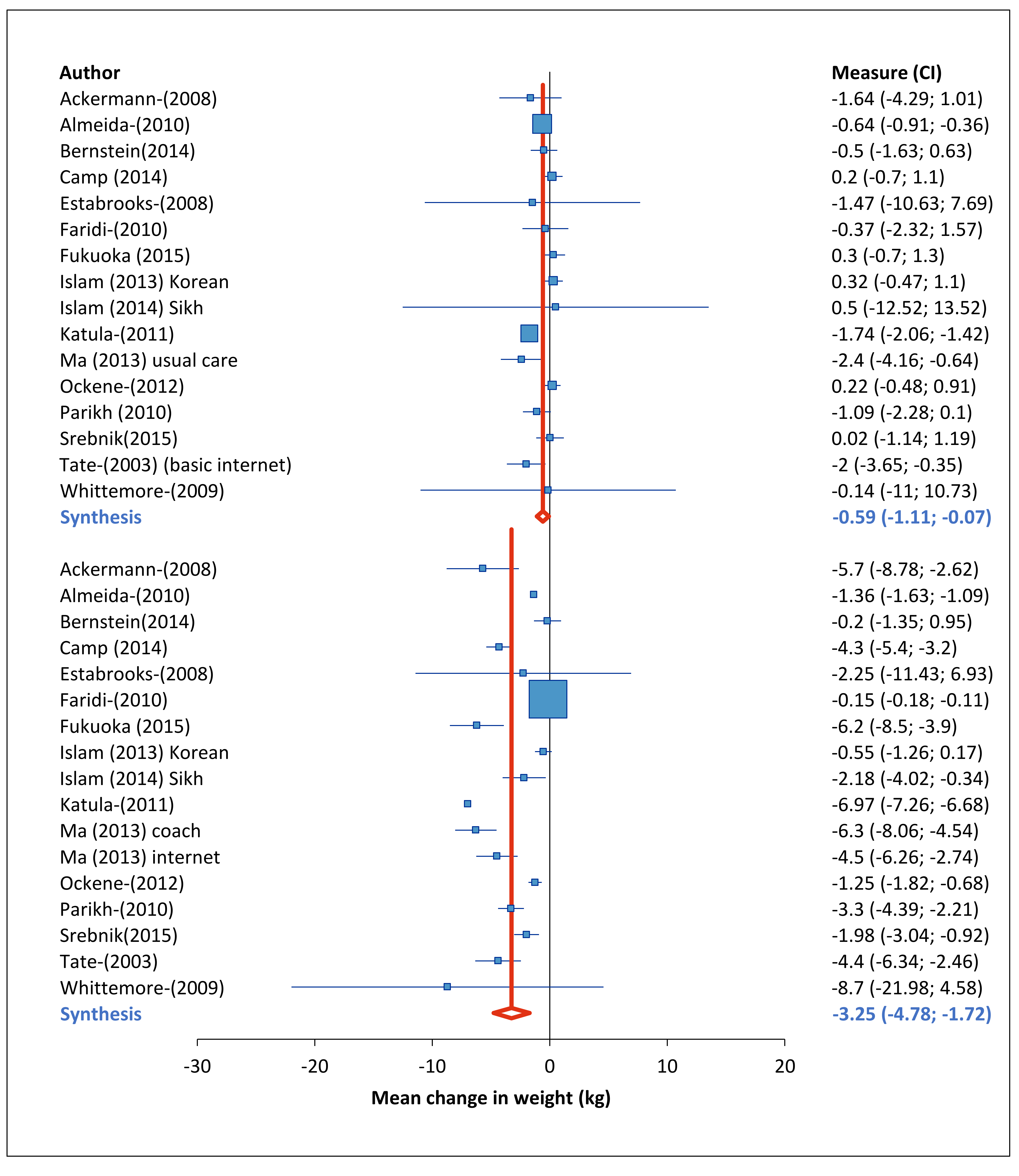

Supplement: S13 Fig — Forest plot of weight change in all studies that reported data for participants in an intervention and a control arm. Listed first are data from the control group participants, and second are data from their respective studies with a duration of 12 mo or greater. (TIFF) [file pmed.1002095.s014.tiff]
