# Supplementary material for: Cardiometabolic Risk Factor Changes Observed in Diabetes Prevention Programs in US Settings: A Systematic Review and Meta-analysis
Source: PLoS Med. 2016 Jul 26;13(7):e1002095. doi: 10.1371/journal.pmed.1002095 (PMC4961455; doi:10.1371/journal.pmed.1002095)
Supplement: S1 Flow Diagram — This flow diagram includes all the records that were identified at each stage of the search with the PRISMA Flow Diagram template. After the application of inclusion and exclusion criteria, 44 studies met criteria and were included in the final analysis. (DOC) [file pmed.1002095.s015.doc]

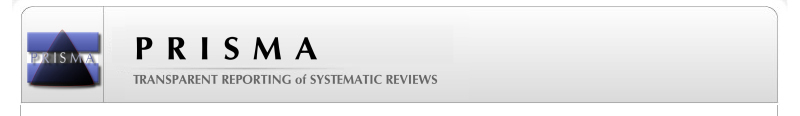
**, PRISMA 2009 Flow Diagram**

**Screening**

**Included**

**Eligibility**

**Identification**

Records identified through database searching
(n = 9,043 )

Additional records identified through other sources
(n = 2 )

Records after duplicates removed
(n = 8,808 )

Records screened
(n = 8,808)

Records excluded
(n = 8,669 )

Full-text articles assessed for eligibility
(n =139 )

Full-text articles excluded
(n = 95 )

with reasons

- Not DPP based intervention
- Outside US
- Lacking relevant follow up data
- Did not include target study population
- Duplicate study or study population

Studies included in qualitative synthesis
(n =44 )

Studies included in quantitative synthesis (meta-analysis)
(n = 44 )
