# Supplementary material for: Cardiometabolic Risk Factor Changes Observed in Diabetes Prevention Programs in US Settings: A Systematic Review and Meta-analysis
Source: PLoS Med. 2016 Jul 26;13(7):e1002095. doi: 10.1371/journal.pmed.1002095 (PMC4961455; doi:10.1371/journal.pmed.1002095)
Supplement: S1 Table — (DOCX) [file pmed.1002095.s016.docx]

**Supplementary Table 1: List of search terms**

| **Cochrane Database (April 2011 to May 2016)**  **673 studies** | **ClinicalTrials.gov (April 2011 to May 2016)**  **100 studies** |
| --- | --- |
| #1 "Overweight/prevention & control"[Mesh]  #2 "Obesity/prevention & control"[Mesh]  #3 "Glucose Intolerance"[Mesh]  #4 "Prediabetic State"[Mesh]  #5 "Diabetes Mellitus/prevention & control" [Mesh]  #6 "Diabetes Mellitus, Type 2/prevention & control"[Mesh]  #7 "Prediabetic State/prevention & control"[Mesh]  #8 "Metabolic Syndrome X/prevention & control"[Mesh]  #9 "Prediabetic State/therapy"[Mesh]  #10 "Diabetes Prevention"  #11 "Diabetes risk reduction"  #12 OR / 1 -11  #13 Weight Loss  #14 Lifestyle  #15 OR/ 13-14  #18 12 AND 15  Limits: English, Humans, Publication Date from 2011 to 2016, product type: trials | #1 overweight prevention  #2 obesity prevention  #3 glucose intolerance  #4 impaired glucose tolerance  #5 impaired fasting glucose  #6 impaired fasting glycemia  #7 metabolic syndrome prevention  #8 diabetes prevention  #9 diabetes risk reduction  Limits: Studies with results, Interventional Studies, English, adults/seniors |

**Supplementary Table 1 (continued)**

| **Medline via PubMed (April 2011 to May 2016)**  **4,653 studies** | **EMBASE (April 2011 to May 2016)**  **3,617studies** |
| --- | --- |
| #1 "Overweight/prevention & control"[Mesh]  #2 "Obesity/prevention & control"[Mesh]  #3 "Glucose Intolerance"[Mesh]  #4 "Prediabetic State"[Mesh]  #5 "Diabetes Mellitus/prevention & control" [Mesh]  #6 "Diabetes Mellitus, Type 2/prevention & control"[Mesh]  #7 "Prediabetic State/prevention & control"[Mesh]  #8 "Metabolic Syndrome X/prevention & control"[Mesh]  #9 "Prediabetic State/therapy"[Mesh]  #10 "Diabetes Prevention"  #11 "Diabetes risk reduction"  #12 OR / 1 -11  #13 Weight Loss  #14 Lifestyle  #15 Preventive Health Services  #16 Program evaluation  #17 OR/ 13-10  #18 12 AND 18  Limits: English, Humans, Publication Date from 2011/04/01 to 2013/04/01 | #1 overweight prevention  #2 obesity prevention  #3 glucose intolerance  #4 impaired glucose tolerance  #5 impaired fasting glucose  #6 impaired fasting glycemia  #7 metabolic syndrome prevention  #8 diabetes prevention  #9 diabetes risk reduction  # 10 OR/ 1-9  #11 weight loss  #12 lifestyle  # 13 OR/ 11 12  #14 10 AND 13  Limits: English, Publication Date from 2011 to 2016/05/01 |
