# Supplementary material for: Cardiometabolic Risk Factor Changes Observed in Diabetes Prevention Programs in US Settings: A Systematic Review and Meta-analysis
Source: PLoS Med. 2016 Jul 26;13(7):e1002095. doi: 10.1371/journal.pmed.1002095 (PMC4961455; doi:10.1371/journal.pmed.1002095)
Supplement: S2 Table — (DOCX) [file pmed.1002095.s017.docx]

**Supplementary Table 2: Baseline participant and intervention characteristics by study**

|  |  | **Patient Characteristics** | | | | | **Intervention Characteristics** | | | | **Article characteristics** |
| --- | --- | --- | --- | --- | --- | --- | --- | --- | --- | --- | --- |
| **Study** | **Study name (year) group name** | **Age**  **(years)** | **n** | **Male %** | **NHW %** | **Baseline BMI** | **Core Sessions** | **Maintenance**  **Yes (y) or no (n)** | **Follow up time (in months)** | **Session type: individual (i) group (g) or both (b)** | **Quality score classification**  **(< 2 points = 0**  **2+ points = 1)** |
| 1 | Ackermann(2008)^1^ | 56.5 | 46 | 50.0 | 93.0 | 32.00 | 16 | y | 12 | g | 1 |
| 2 | Ackermann(2015)^2^ | 50.8 | 257 | 27.2 | 35.4 | 37.10 | 16 | Y | 12 | g | 1 |
| 3 | Aldana(2005)^3^ | 46.0 | 37 | 34.3 | 48.6 | 32.01 | 16 | Y | 12 | g | 1 |
| 4 | Almeida(2010)^4^ | 62.4 | 820 | 48.0 | 70.0 | 29.80 | 1 | n | 12 | g | 0 |
| 5 | Bernstein (2014) ^5^ | - | 14 | 0.0 | 0.0 | 37.20 | 6 | N | 1.5 | g | 0 |
| 6 | Boltri(2008)^6^ | 52.0 | 8 | 42.0 | 0.0 | 31.60 | 16 | n | 12 | g | 1 |
| 7 | Boltri(2011)^7^ | 57.2 | 37 | 30.0 | 0.0 | 33.20 | 12 | y | 12 | g | 1 |
| 8 | Camp(2014)^8^ | 42.2 | 109 | 0.0 | - | 35.80 | 14 | n | 12 | g | 1 |
| 9 | DavisSmith(2007)^9^ | 55.9 | 10 | 30.0 | 0.0 | 35.70 | 6 | n | 12 | g | 1 |
| 10 | Estabrooks(2008)^10^ | 57.8 | 39 | 28.2 | 69.0 | - | 12 | n | 3 | i | 1 |
| 11 | Faridi(2010)^11^ | 49.0 | 121 | 15.0 | 0.0 | 33.80 | variable | n | 12 | i | 1 |
| 12 | Fukuoka^12^ | 57.1 | 30 | 23.3 | 43.3 | 32.20 | 6 | N | 5 | b | 1 |
| 13 | Harwell (2011)^13^ | - | 989 | 19.0 | - | - | 16 | y | 10 | g | 0 |
| 14 | Islam (Korean) ^14^ | 61.0 | 25 | 32 | 0 | 24.10 | 6 | Y | 6 | b | 1 |
| 15 | Islam (Sikh) ^15^ | 46.3 | 76 | 4.9 | 0 | 28.20 | 6 | Y | 6 | b | 1 |
| 16 | Jaber(2010)^16^ | 47.0 | 71 | 27.0 | 0.0 | 34.30 | 12 | y | 6 | g | 1 |
| 17 | Jiang (2013)^17^ | 46.6 | 2553 | 25.5 | 0.0 | 35.80 | 16 | y | 36 | g | 1 |
| 18 | Katula(2011)^18^ | 57.3 | 151 | 43.0 | 73.5 | 32.81 | 24 | y | 12 | g | 1 |
| 19a | Kramer(2009) phase 1^19^ | 52.9 | 51 | 18.0 | 73.0 | 36.60 | 12 | n | 3 | g | 0 |
| 19b | Kramer(2009) phase 2^19^ | 57.2 | 42 | 21.0 | 100.0 | 34.60 | 12 | y | 12 | g | 0 |
| 20a | Kramer(2010) DVD^20^ | 57.3 | 22 | 29.0 | 83.0 | 32.85 | 12 | n | 3 | i | 1 |
| 20b | Kramer(2010)GROUP^20^ | 61.0 | 26 | 29.0 | 83.0 | 35.09 | 12 | n | 3 | g | 1 |
| 21 | Kramer(2011)^21^ | 53.0 | 81 | 12.3 | 96.0 | 37.10 | 12 | y | 3 | g | 1 |
| 22a | Ma (2013) coach^22^ | 54.6 | 79 | 51.9 | 77.2 | 31.80 | 12 | y | 15 | g | 1 |
| 22b | Ma (2013) internet^22^ | 51.8 | 81 | 44.3 | 79.0 | 31.70 | 24 | y | 15 | i | 1 |
| 23 | Matvienko(2009)^23^ | 55.7 | 31 | 39.0 | 94.0 | 36.10 | 16 | y | 12 | i | 0 |
| 24 | Mau(2010)^24^ | 49.0 | 239 | 17.0 | 0.0 | 39.10 | 8 | n | 3 | g | 0 |
| 25 | McBride(2008)^25^ | 51.9 | 40 | 41.0 | 100.0 | 37.40 | 12 | y | 12 | g | 1 |
| 26 | McTigue(2009) internet^26^ | 51.9 | 50 | 24.0 | 86.0 | 36.40 | 16 | y | 12 | g | 1 |
| 27 | McTigue(2009) Willow^27^ | 53.0 | 81 | 17.0 | 86.0 | 38.89 | 12 | y | 12 | g | 1 |
| 28 | Ockene(2012)^28^ | 52.0 | 162 | 28.0 | 0.0 | 33.57 | 16 | n | 12 | b | 1 |
| 29 | Pagoto(2008)^29^ | 48.7 | 118 | 28.0 | 90.7 | 43.30 | 16 | y | 4 | g | 0 |
| 30 | Parikh(2010)^30^ | 46.0 | 50 | 14.0 | 2.0 | 32.00 | 8 | n | 12 | g | 1 |
| 31 | Ruggiero (2011)^31^ | 37.9 | 69 | 7.2 | 0.0 | 31.19 | 16 | y | 12 | g | 0 |
| 32 | Sattin (2014)^32^ | 46.5 | 604 | 16.6 | 0.0 | 35.70 | 16 | y | 12 | b | 1 |
| 33 | Seidel(2008)^33^ | 54.0 | 88 | 15.9 | 72.7 | 36.20 | 12 | n | 6 | g | 1 |
| 34 | Sepah (2014)^34^ | 43.9 | 187 | 15.1 | 51.1 | 36.70 | 16 | y | 12 | i | 1 |
| 35 | Srebnik^35^ | 51.0 | 60 | 35 | 62 | 36.70 | 16 | Y | 12 | g | 1 |
| 36 | Swanson (2012)^36^ | 62.0 | 221 | 33.0 | 88.0 | 31.20 | 4 | n | 6 | g | 1 |
| 37 | Tang^37^ | 60.0 | 13 | 27 | 0 | - | 6 | Y | 5 | b | 0 |
| 38 | Tate(2003)^38^ | 49.8 | 46 | 9.0 | 89.0 | 32.50 | 5 | y | 12 | i | 1 |
| 39 | Vadheim(2010)^39^ | 50.5 | 101 | 12.0 | 92.5 | 36.20 | 16 | y | 10 | g | 0 |
| 40a | Vadheim(2010) onsite^40^ | 53.0 | 13 | 31.0 | - | 34.00 | 16 | y | 4 | g | 1 |
| 40b | Vadheim(2010) telehealth^40^ | 50.0 | 14 | 7.0 | - | 38.70 | 16 | y | 4 | g | 1 |
| 41 | Vanderwood(2010)^41^ | 52.3 | 1003 | 20.0 | 92.5 | 35.10 | 16 | y | 10 | g | 0 |
| 42 | Vincent^42^ | 50.0 | 38 | 23.7 | 0 | 34.60 | 8 | Y | 5 | g | 0 |
| 43 | Whittemore(2009)^43^ | 48.2 | 31 | 10.0 | 48.0 | 40.00 | 11 | n | 6 | i | 1 |
| 44 | Yeary(2011)^44^ | 50.8 | 26 | 15.0 | 0.0 | 35.00 | 16 | n | 4 | g | 1 |

*If a study presented two intervention groups, then the study number is listed twice with an a or b to designate the group.

**References**

1. Ackermann RT, Finch EA, Caffrey HM, Hays LM, Saha C. Successful implementation of a large, community-based comparative effectiveness trial of the Diabetes Prevention Program (DPP). Diabetes 2011;60:A11.

2. Ackermann RT, Liss DT, Finch EA, et al. A Randomized Comparative Effectiveness Trial for Preventing Type 2 Diabetes. American journal of public health 2015;105:2328-34.

3. Aldana SG, Barlow M, Smith R, et al. The diabetes prevention program: a worksite experience. AAOHN J 2005;53:499-505; quiz 6-7.

4. Almeida FA, Shetterly S, Smith-Ray RL, Estabrooks PA. Reach and effectiveness of a weight loss intervention in patients with prediabetes in Colorado. Prev Chronic Dis 2010;7:A103.

5. Bernstein AM, Gendy G, Rudd N, et al. Management of prediabetes through lifestyle modification in overweight and obese African-American women: the Fitness, Relaxation, and Eating to Stay Healthy (FRESH) randomized controlled trial. Public health 2014;128:674-7.

6. Boltri JM, Davis-Smith YM, Seale JP, Shellenberger S, Okosun IS, Cornelius ME. Diabetes prevention in a faith-based setting: results of translational research. J Public Health Manag Pract 2008;14:29-32.

7. Boltri JM, Davis-Smith M, Okosun IS, Seale JP, Foster B. Translation of the National Institutes of Health Diabetes Prevention Program in African American churches. J Natl Med Assoc 2011;103:194-202.

8. Camp A, Tamborlane WV, Kim G, Magenheimer E, Montosa A. Successful implementation of a T2D prevention program in a community health center setting. Diabetes 2013;62:A330.

9. Davis-Smith YM, Boltri JM, Seale JP, Shellenberger S, Blalock T, Tobin B. Implementing a diabetes prevention program in a rural African-American church. J Natl Med Assoc 2007;99:440-6.

10. Estabrooks PA, Smith-Ray RL. Piloting a behavioral intervention delivered through interactive voice response telephone messages to promote weight loss in a pre-diabetic population. Patient Educ Couns 2008;72:34-41.

11. Faridi Z, Shuval K, Njike VY, et al. Partners reducing effects of diabetes (PREDICT): a diabetes prevention physical activity and dietary intervention through African-American churches. Health Educ Res 2010;25:306-15.

12. Fukuoka Y, Gay CL, Joiner KL, Vittinghoff E. A Novel Diabetes Prevention Intervention Using a Mobile App: A Randomized Controlled Trial With Overweight Adults at Risk. Am J Prev Med 2015;49:223-37.

13. Harwell TS, Vanderwood KK, Hall TO, Butcher MK, Helgerson SD. Factors associated with achieving a weight loss goal among participants in an adapted Diabetes Prevention Program. Prim Care Diabetes 2011;5:125-9.

14. Islam NS, Zanowiak JM, Wyatt LC, et al. A randomized-controlled, pilot intervention on diabetes prevention and healthy lifestyles in the New York City Korean community. Journal of community health 2013;38:1030-41.

15. Islam NS, Zanowiak JM, Wyatt LC, et al. Diabetes prevention in the New York City Sikh Asian Indian community: a pilot study. International journal of environmental research and public health 2014;11:5462-86.

16. Jaber LA, Pinelli NR, Brown MB, et al. Feasibility of group lifestyle intervention for diabetes prevention in Arab Americans. Diabetes Res Clin Pract 2011;91:307-15.

17. Jiang L, Manson SM, Beals J, et al. Translating the Diabetes Prevention Program into American Indian and Alaska Native communities: results from the Special Diabetes Program for Indians Diabetes Prevention demonstration project. Diabetes care 2013;36:2027-34.

18. Katula JA, Vitolins MZ, Rosenberger EL, et al. One-year results of a community-based translation of the Diabetes Prevention Program: Healthy-Living Partnerships to Prevent Diabetes (HELP PD) Project. Diabetes care2011:1451-7.

19. Kramer MK, Kriska AM, Venditti EM, et al. Translating the Diabetes Prevention Program: a comprehensive model for prevention training and program delivery. Am J Prev Med 2009;37:505-11.

20. Kramer MK, Kriska AM, Venditti EM, et al. A novel approach to diabetes prevention: evaluation of the Group Lifestyle Balance program delivered via DVD. Diabetes Res Clin Pract 2010;90:e60-3.

21. Kramer MK, McWilliams JR, Chen HY, Siminerio LM. A community-based diabetes prevention program: evaluation of the group lifestyle balance program delivered by diabetes educators. Diabetes Educ 2011;37:659-68.

22. Ma J, Yank V, Xiao L, et al. Translating the Diabetes Prevention Program lifestyle intervention for weight loss into primary care: a randomized trial. JAMA Intern Med 2013;173:113-21.

23. Matvienko OA, Hoehns JD. A lifestyle intervention study in patients with diabetes or impaired glucose tolerance: translation of a research intervention into practice. Journal of the American Board of Family Medicine : JABFM 2009;22:535-43.

24. Mau MK, Keawe'aimoku Kaholokula J, West MR, et al. Translating diabetes prevention into native Hawaiian and Pacific Islander communities: the PILI 'Ohana Pilot project. Prog Community Health Partnersh 2010;4:7-16.

25. McBride PE, Einerson JA, Grant H, et al. Putting the Diabetes Prevention Program into practice: a program for weight loss and cardiovascular risk reduction for patients with metabolic syndrome or type 2 diabetes mellitus. J Nutr Health Aging 2008;12:745S-9S.

26. McTigue KM, Conroy MB, Hess R, et al. Using the internet to translate an evidence-based lifestyle intervention into practice. Telemed J E Health 2009;15:851-8.

27. McTigue KM, Conroy MB, Bigi L, Murphy C, McNeil M. Weight loss through living well: translating an effective lifestyle intervention into clinical practice. Diabetes Educ 2009;35:199-204, 8.

28. Ockene IS, Tellez TL, Rosal MC, et al. Outcomes of a Latino community-based intervention for the prevention of diabetes: the Lawrence Latino Diabetes Prevention Project. American journal of public health 2012;102:336-42.

29. Pagoto SL, Kantor L, Bodenlos JS, Gitkind M, Ma Y. Translating the diabetes prevention program into a hospital-based weight loss program. Health Psychol 2008;27:S91-8.

30. Parikh P, Simon EP, Fei K, Looker H, Goytia C, Horowitz CR. Results of a pilot diabetes prevention intervention in East Harlem, New York City: Project HEED. American journal of public health 2010;100 Suppl 1:S232-9.

31. Ruggiero L, Oros S, Choi YK. Community-based translation of the diabetes prevention program's lifestyle intervention in an underserved Latino population. Diabetes Educ 2011;37:564-72.

32. Sattin RW, Dias JK, Williams LB, Joshua T, Marion LN. Effects on weight of a cluster-randomized, controlled trial of a faith-based adaption of the diabetes prevention program within African-American churches. Diabetes 2014;63:A526-A7.

33. Seidel MC, Powell RO, Zgibor JC, Siminerio LM, Piatt GA. Translating the Diabetes Prevention Program into an urban medically underserved community: a nonrandomized prospective intervention study. Diabetes care 2008;31:684-9.

34. Sepah SC, Jiang L, Peters AL. Translating the Diabetes Prevention Program into an Online Social Network: Validation against CDC Standards. Diabetes Educ 2014;40:435-43.

35. Srebnik D, Chwastiak LA, Russo J, Sylla L. A pilot study of the diabetes prevention program on weight loss for adults at community mental health centers. Psychiatric services 2015;66:200-3.

36. Swanson CM, Bersoux S, Larson MH, et al. An outpatient-based clinical program for diabetes prevention: an update. Endocr Pract 2012;18:200-8.

37. Tang TS, Nwankwo R, Whiten Y, Oney C. Outcomes of a church-based diabetes prevention program delivered by peers: a feasibility study. Diabetes Educ 2014;40:223-30.

38. Tate DF, Jackvony EH, Wing RR. Effects of Internet behavioral counseling on weight loss in adults at risk for type 2 diabetes: a randomized trial. JAMA 2003;289:1833-6.

39. Vadheim LM, Brewer KA, Kassner DR, et al. Effectiveness of a lifestyle intervention program among persons at high risk for cardiovascular disease and diabetes in a rural community. J Rural Health 2010;26:266-72.

40. Vadheim LM, McPherson C, Kassner DR, et al. Adapted diabetes prevention program lifestyle intervention can be effectively delivered through telehealth. Diabetes Educ 2010;36:651-6.

41. Vanderwood KK, Hall TO, Harwell TS, et al. Implementing a state-based cardiovascular disease and diabetes prevention program. Diabetes care 2010;33:2543-5.

42. Vincent D, McEwen MM, Hepworth JT, Stump CS. The effects of a community-based, culturally tailored diabetes prevention intervention for high-risk adults of Mexican descent. Diabetes Educ 2014;40:202-13.

43. Whittemore R, Melkus G, Wagner J, Dziura J, Northrup V, Grey M. Translating the diabetes prevention program to primary care: a pilot study. Nurs Res 2009;58:2-12.

44. Yeary KH, Cornell CE, Turner J, et al. Feasibility of an evidence-based weight loss intervention for a faith-based, rural, African American population. Prev Chronic Dis 2011;8:A146.
