# Supplementary material for: Cardiometabolic Risk Factor Changes Observed in Diabetes Prevention Programs in US Settings: A Systematic Review and Meta-analysis
Source: PLoS Med. 2016 Jul 26;13(7):e1002095. doi: 10.1371/journal.pmed.1002095 (PMC4961455; doi:10.1371/journal.pmed.1002095)
Supplement: S3 Table — (DOCX) [file pmed.1002095.s018.docx]

**Supplementary Table 3:** Scoring system adapted from the Juni scoring criteria

**Method of quality assessment.** To qualify as “high quality” needed 2 or more of the following:

| Criterion 1: Methods to minimize bias  Any of the following | - Used an intention to treat analysis - Achieved an attrition rate < 20% - Compared characteristics of completers and non-completers |
| --- | --- |
| Criterion 2: Methods clearly describe translation  Included 4+ of the following | - description of the process of designing the program - description of the enrollment process - documentation of session attendance, reporting costs and/or resource inputs - documentation of the training process or qualifications of personnel - description of the qualitative feedback from participants or providers |
| Criterion 3: presence of a control group | - had an arm of randomized, matched, or unmatched comparison |
